# Supplementary material for: Flavonoids and Terpenoids with PTP-1B Inhibitory Properties from the Infusion of Salvia amarissima Ortega
Source: Molecules. 2020 Aug 1;25(15):3530. doi: 10.3390/molecules25153530 (PMC7435600; doi:10.3390/molecules25153530)
Supplement: Supplementary file 1 [file molecules-25-03530-s001.pdf]

SUPPLEMENTARY MATERIALS

Flavonoids and Terpenoids with PTP-1B Inhibitory Properties from the Infusion of *Salvia amarissima* Ortega#

Eric Salinas-Arellano[1,#], Araceli Pérez-Vásquez[1], Isabel Rivero-Cruz[1], Rafael Torres-Colin[2], Martin González Andrade[3], Manuel Rangel-Grimaldo[1] and Rachel Mata[1,*]

[1] Facultad de Química, Universidad Nacional Autónoma de México, Mexico City 04510, Mexico; ersalinass@hotmail.com (E.A.-S.); perezva@unam.mx (A.P.-V.); riveroic@unam.mx (I.R.-C.); manuel_erg_p9@hotmail.com (M.R-G.); rachel@unam.mx (R.M.)

[2] Instituto de Biología, Universidad Nacional Autónoma de México, Mexico City 04510, Mexico; rafael.torres@ib.unam.mx (R.T.-C.)

[3] Facultad de Medicina, Universidad Nacional Autónoma de México, Mexico City 04510, Mexico; martin@bq.unam.mx (M.G.A.)

* Correspondence: rachel@unam.mx; Tel.: + 52-55-56225289

#Taken in part from the PhD thesis of Eric Salinas-Arellano

Contents

Spectroscopic and spectrometric data of compounds (**1–6**)
Figure S1. ^{1}H NMR spectrum of Amarisolide (compound **1**, 400 MHz, CD_3OD)
Figure S2. ^{13}C NMR spectrum of Amarisolide (compound **1**, 100 MHz, CD_3OD)
Figure S3. ^{1}H NMR spectrum of 5,6,4'-Trihidroxy-7,3'-dimethoxyflavone (compound **2**, 700 MHz, CD_3OD)
Figure S4. ^{13}C NMR spectrum of 5,6,4'-Trihidroxy-7,3'-dimethoxyflavone (compound **2**, 175 MHz, CD_3OD)
Figure S5. ^{1}H NMR spectrum of 6-Hydroxyluteolin (compound **3**, 700 MHz, CD_3OD)
Figure S6. ^{13}C NMR spectra of 6-Hydroxyluteolin (compound **3**, 175 MHz, CD_3OD)
Figure S7. ^{1}H NMR spectrum of Rutin (compound **4**, 400 MHz, CD_3OD)
Figure S8. ^{1}H NMR spectrum of Rosmarinic acid (compound **5**, 700 MHz, CD_3OD)
Figure S9. ^{13}C NMR spectrum of Rosmarinic acid (compound **5**, 175 MHz, CD_3OD)
Figure S10. ^{1}H NMR spectrum of Isoquercitrin (compound **6**, 700 MHz, CD_3OD)
Figure S11. ^{13}C NMR spectrum of Isoquercitrin (compound **6**, 175 MHz, CD_3OD)
Figure S12. IR spectrum of Amarisolide G (compound **8a**,**b**)
Figure S13. ^{1}H NMR spectrum of Amarisolide G (compound **8a**,**b**, 700 MHz, DMSO-d_6)
Figure S14. ^{13}C NMR spectrum of Amarisolide G (compound **8a**,**b**, 175 MHz, DMSO-d_6)
Figure S15. ^{1}H-^{1}H COSY spectrum of Amarisolide G (compound **8a**,**b**)
Figure S16. ^{1}H-^{13}C HSQC spectrum of Amarisolide G (compound **8a**,**b**)
Figure S17. ^{1}H-^{13}C HMBC spectrum of Amarisolide G (compound **8a**,**b**)
Figure S18. TOCSY spectrum of Amarisolide G (compound **8a**,**b**)
Figure S19. Total ion current chromatogram of the essential oil of *S. amarissima* (Sa-Batch 1). Peak identification: **10**, (*E*)-Pinocarvyl acetate; **11**, δ-elemene; **12**, α-bourbonene; **13**, β-caryophyllene; **14**, α-caryophyllene; **15**, germacrene D; **16**, β-selinene; **17**, spathulenol.
Figure S20. Total ion current chromatogram of the essential oil of *S. amarissima* (Sa-Batch 1). Peak identification: **9**, 3-Methoxy-*p*-cymene; **10**, (*E*)-pinocarvyl acetate; **12**, α-bourbonene; **13**, β-caryophyllene; **14**, α-caryophyllene; **15**, germacrene D; **16**, β-selinene; **17**, spathulenol.

Figure S21. EIMS spectrum of 3-Methoxy-*p*-cymene (compound **9**)
Figure S22. EIMS spectrum of (*E*)-Pinocarvyl acetate (compound **10**)
Figure S23. EIMS spectrum of δ-Elemene (compound **11**)
Figure S24. EIMS spectrum of α-Bourbonene (compound **12**)
Figure S25. EIMS spectrum of β-Caryophyllene (compound **13**)
Figure S26. EIMS spectrum of α-Caryophyllene (compound **14**)
Figure S27. EIMS spectrum of Germacrene D (compound **15**)
Figure S28. EIMS spectrum of β-Selinene (compound **16**)
Figure S29. EIMS spectrum of Spathulenol (compound **17**)
Figure S30. Residues of interaction to 4 Å of compounds **1,7** and **UA** at the catalytic site of PTP1B.
Figure S31. Residues of interaction to 4 Å of compounds **1, 7** and **UA** at the allosteric site of PTP1B.

Spectroscopic and spectrometric data of compounds (**1–7**)

*Amarisolide (**1**)*. ^{1}H-NMR (400 MHz, CD_3OD) δ_H (ppm): 7.35 (dd, J = 1.7 Hz, H-15), 7.28 (dd, J = 1.3 Hz, H-16), 6.76 (d, J = 6.3 Hz, H-3), 6.33 (dd, J = 1.8, 0.9 Hz, H-14), 4.62 (dt, J = 6.4, 2.2 Hz, H-2), 4.46 (d, J = 7.7 Hz, H-1'), 4.41 (d, J = 8.2 Hz, H-19a), 4.02 (dd, J = 8.2, 2.0 Hz, H-19b), 3.83 (dd, J =11.8, 2.3 Hz, H-6'a), 3.51 (dd, J = 11.8, 6.6 Hz, H-6'b), 3.33 (d, J = 9.0 Hz, H-3'), 3.27 (m, H-5'), 3.14 (m, H-2', H-4'), 2.64 (td, J = 13.6, 4.4 Hz, H-12a), 2.47 (d, J = 13.4 Hz, H-10), 2.33 (td, J = 13.5, 5.0 Hz, H-12b), 1.86 (m, H-6a), 1.79 (ddd, J = 12.2, 8.9, 4.8 Hz, H-1a), 1.63 (m, H-7), 1.61 (m, H-8), 1.41 (m, H-11), 1.37 (m, H-1b), 1.30 (m, H-6b), 0.88 (d, J = 6.7 Hz, H-17), 0.61 (s, H-20); ^{13}C-NMR (100 MHz, CD_3OD) δ_C (ppm): 170.2 (C-18), 143.3 (C-4), 142.4 (C-15), 138.3 (C-16), 130.3 (C-3), 125.7 (C-13), 110.8 (C-14), 101.9 (C-1'), 76.9 (C-5'), 76.7 (C-3'), 74.1 (C-2'), 71.2 (C-19), 70.5 (C-4'), 69.7 (C-2), 61.6 (C-6'), 45.7 (C-5), 39.6 (C-10), 37.8 (C-8,C-9), 33.5 (C-6), 27.4 (C-7), 26.1 (C-1), 26.0 (C-11), 17.0 (C-12), 16.6 (C-20), 14.5 (C-17).

*5,6,4'-Trihydroxy-7,3'-dimethoxyflavone (**2**)*. ^{1}H-NMR (700 MHz, CD_3OD) δ_H (ppm): 7.59 (dd, J = 8.3, 2.2 Hz, H-6'), 7.57 (d, J =2.1 Hz, H-2'), 6.98 (d, J = 8.2 Hz, H-5'), 6.91 (s, H-8), 6.71 (s, H-3), 4.04 (s, 7-OCH_3), 4.02 (s, 3'-OCH_3); ^{13}C-NMR (175 MHz, CD_3OD) δ_C (ppm): 182.9 (C-4), 165.1 (C-2), 154.5 (C-7), 150.7 (C-9), 150.6 (C-4'), 148.1 (C-5), 145.8 (C-5'), 130.1 (C-6), 122.4 (C-1'), 120.4 (C-2'), 115.4 (C-3'), 112.6 (C-6'), 105.2 (C-10), 102.5 (C-3), 90.6 (C-8), 55.5 (7-OCH_3), 55.3 (3'-OCH_3) [11].

*6-hydroxyluteolin (**3**)*. ^{1}H-NMR (700 MHz, CD_3OD) δ_H (ppm): 7.42 (dd, J = 8.4, 2.3 Hz, H-6'), 7.40 (d, J = 2.3 Hz, H-2'), 6.88 (d, J = 8.4 Hz, H-5'), 6.83 (s, H-3), 6.58 (s, H-8); ^{13}C-NMR (175 MHz, CD_3OD) δ_C (ppm): 183.9 (C-4), 166.8 (C-2), 155.3 (C-9), 151.5 (C-7,C-4'), 147.7 (C-5), 146.9 (C-3'), 131.2 (C-6), 121.6 (C-1'), 120.1 (C-6'), 116.6 (C-5'), 113.0 (C-2'), 106.1 (C-10), 102.6 (C-3), 91.8 (C-8).

*Rutin (**4**)*. ^{1}H-NMR (400 MHz, CD_3OD) δ_H (ppm): 7.65 (d, J = 2.2 Hz, H-2'), 7.62 (dd, J = 8.4, 2.2 Hz, H-6'), 6.86 (d, J = 8.4 Hz, H-5'), 6.38 (d, J = 2.1 Hz, H-6), 6.19 (d, J = 2.1 Hz, H-8), 5.08 (d, J = 7.6 Hz, H-1''), 4.50 (d, J = 1.7 Hz, H-1'''), 3.79 (dd, J = 11.0, 1.5 Hz, H-5''), 3.61 (dd, J = 3.5, 1.7 Hz, 5'''), 3.35-3.54 (m, H-2'', H-3'', H-4'', H-6'', H-2'''-H-4'''), 1.11 (d, J = 6.2 Hz, 6''').

*Rosmarinic acid (**5**)*. ^{1}H-NMR (700 MHz, CD_3OD) δ_H (ppm): 7.53 (d, J = 15.9 Hz, H-7), 7.05 (d, J = 2.1 Hz, H-2), 6.94 (dd, J = 8.2, 2.1 Hz, H-6), 6.79 (d, J = 8.1 Hz, H-5), 6.78 (d, J = 2.2 Hz, H-2'), 6.70 (d, J = 8.0 Hz, H-5'), 6.65 (dd, J = 8.1, 2.0 Hz, H-6'), 6.29 (d, J =15.8 Hz, H-8), 5.10 (d, J = 8.0 Hz, H-8'), 3.10 (dd, J = 14.3, 3.2 Hz, H-7'a), 2.95 (dd, J = 14.3, 9.5 Hz, H-7'b); ^{13}C-NMR (175 MHz, CD_3OD) δ_C (ppm): 177.0 (C-9'), 169.0(C-9), 149.3 (C-4), 146.6 (C-7), 146.6 (C-3), 145.8 (C-3'), 144.7 (C-4'), 130.8 (C-1'), 127.8 (C-1), 122.8 (C-6), 121.6 (C-6'), 117.4 (C-2'), 116.3 (C-5'), 116.1 (C-5), 115.4 (C-2), 115.0 (C-8), 77.4 (C-8'), 38.5 (C-7'); ESI-MS *m/z* 359.23 $[M-H]^-$.

*Isoquercitrin (**6**)*. ^{1}H-NMR (700 MHz, CD_3OD) δ_H (ppm): 7.73 (d, J = 2.1 Hz, H-2'), 7.60 (dd, J = 8.4, 2.1 Hz, H-6'), 6.88 (dd, J =8.4 Hz, H-5'), 6.35 (d, J = 2.0 Hz, H-8), 6.17 (d, J = 2.0 Hz, H-6), 5.21 (d, J = 7.7 Hz, H-1''), 3.70 (dd, J = 11.9, 2.4 Hz, H-6a''), 3.60 (dd, J = 11.8, 5.2 Hz, H-6b''), 3.50 (dd, J = 9.2, 7.8 Hz, H-3''), 3.44 (t, J = 9.0 Hz, H-5''), 3.24 (m, H-2'', H-4''); ^{13}C-NMR (175 MHz, CD_3OD) δ_C (ppm): 177.7 (C-4), 167.4 (C-7), 161.5 (C-5), 157.3 (C-2), 157.3 (C-9), 148.6 (C-4'), 144.6 (C-3'), 134.1 (C-3), 121.7 (C-6'), 121.7 (C-1'), 116.1 (C-2'), 114.6 (C-5'), 103.5 (C-1''), 103.2 (C-10), 99.4 (C-6), 94.0 (C-8), 77.0 (C-5''), 76.8 (C-2''), 74.3 (C-3''), 69.8 (C-4''), 61.1 (C-6'').

Pedalitin (**7**). Yellow powder; m.p. 300–301 °C; R_T 2.82 min; ESI-MS *m/z* 315.48 $[M-H]^-$ [7].

Figure S1. ^{1}H NMR spectrum of Amarisolide (compound **1**, 400 MHz, CD_3OD)

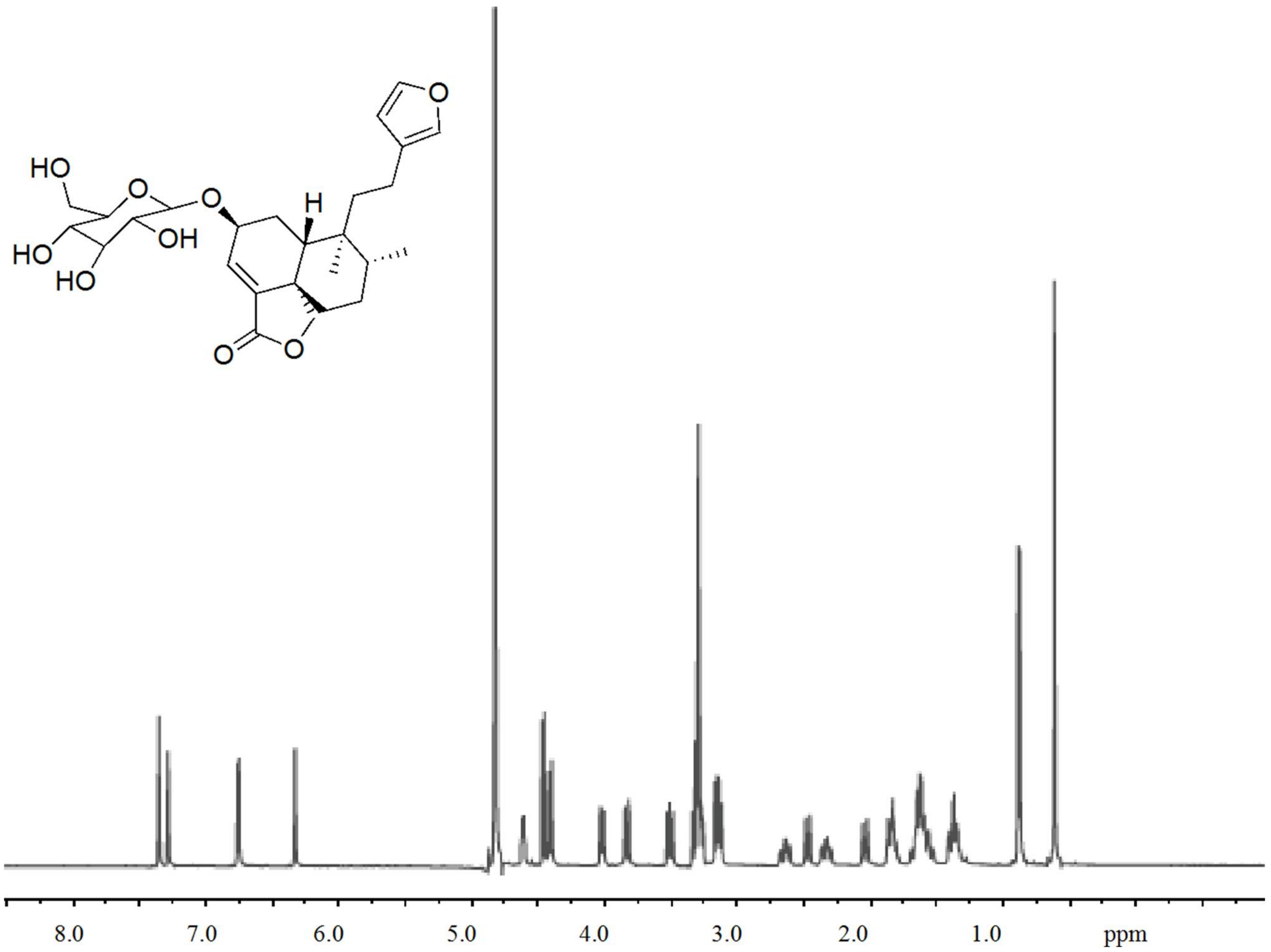

Figure S2. ^{13}C NMR spectrum of Amarisolide (compound **1**, 100 MHz, CD_3OD)

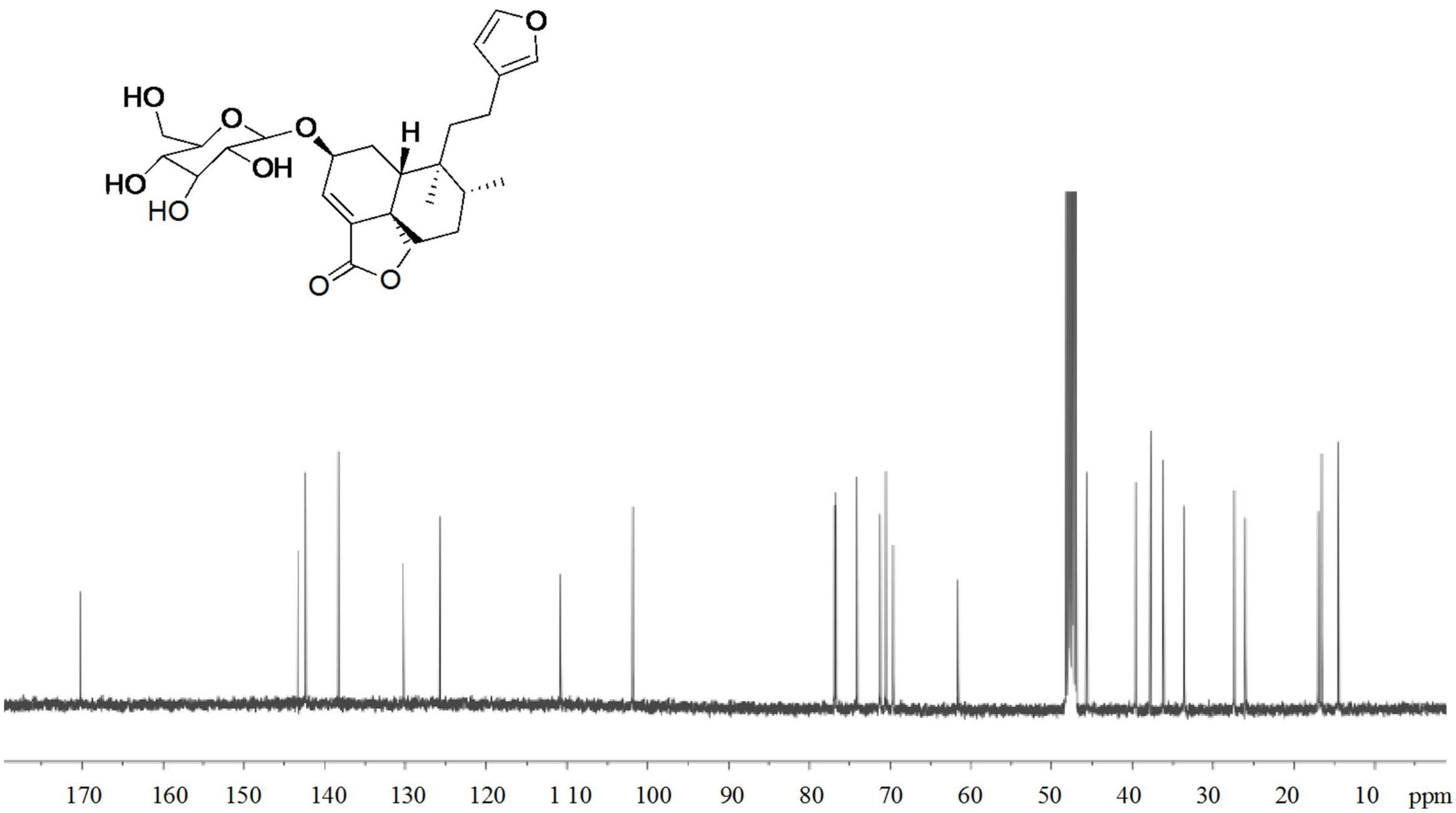

Figure S3. ^{1}H NMR spectrum of 5,6,4'-Trihidroxy-7,3'-dimethoxyflavone (compound **2**, 700 MHz, CD_3OD)

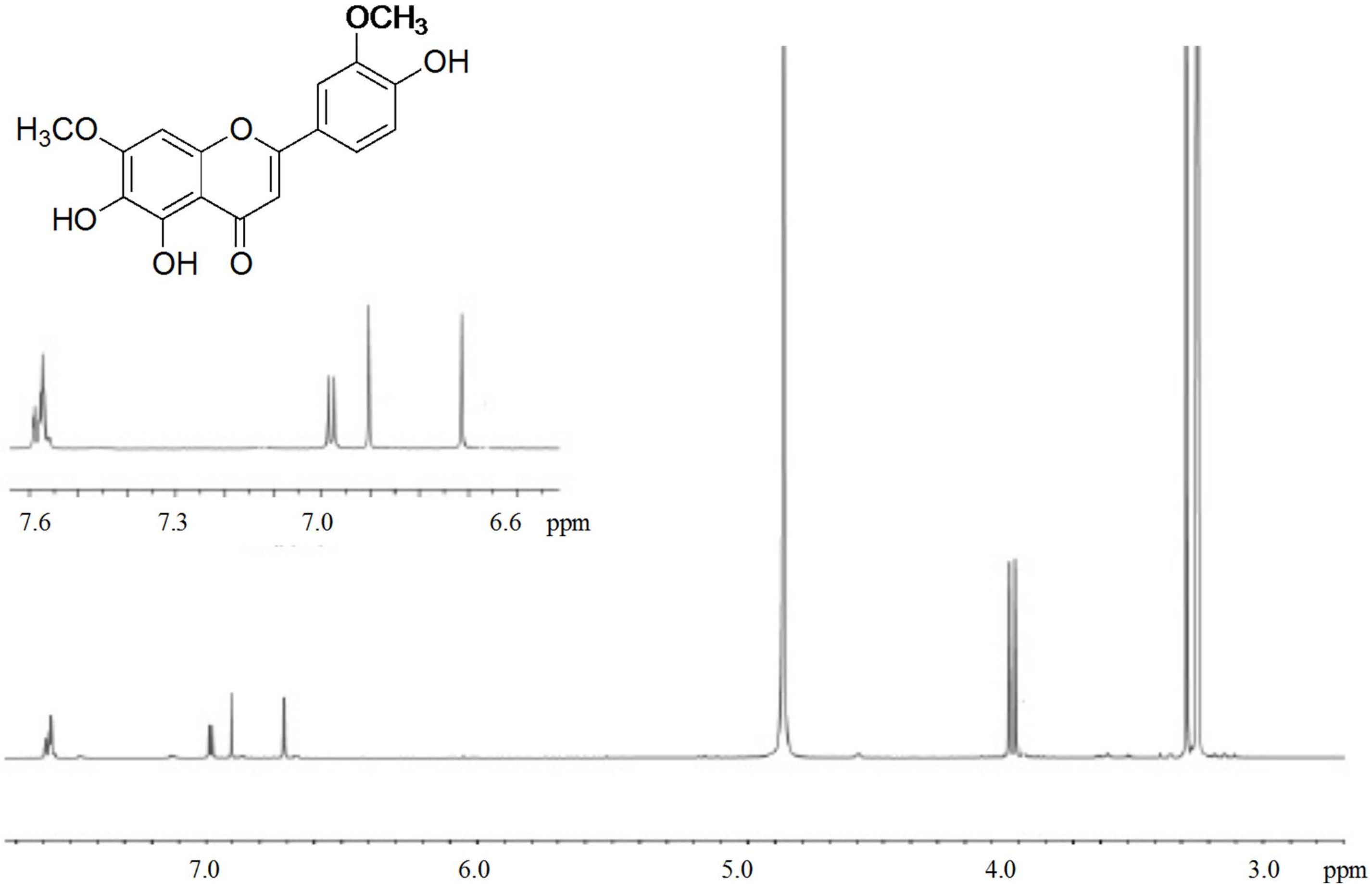

Figure S4. ^{13}C NMR spectrum of 5,6,4’-Trihidroxy-7,3’-dimethoxyflavone (compound **2**, 175 MHz, CD_3OD)

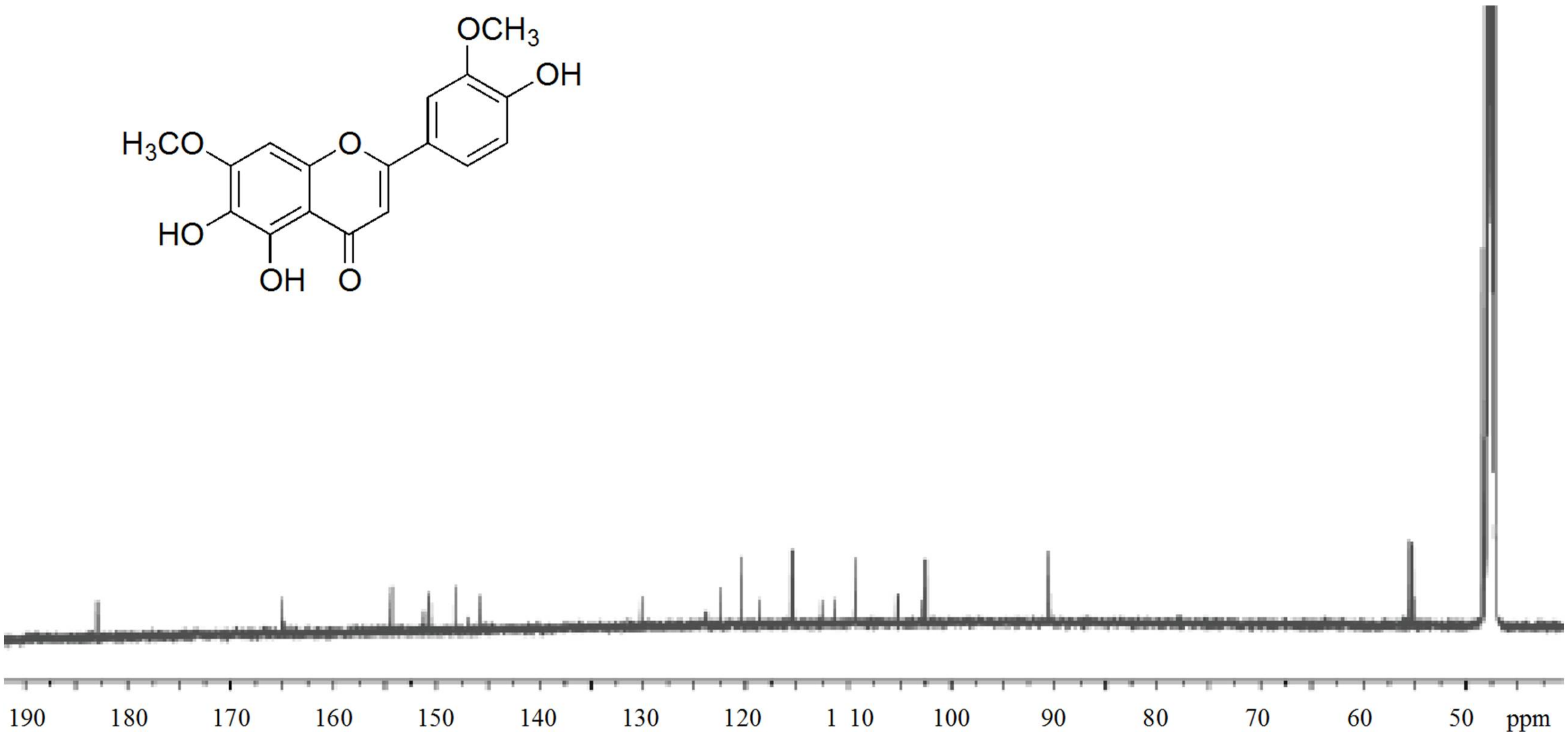

Figure S5. ^{1}H NMR spectrum of 6-Hydroxyluteolin (compound **3**, 700 MHz, CD_3OD)

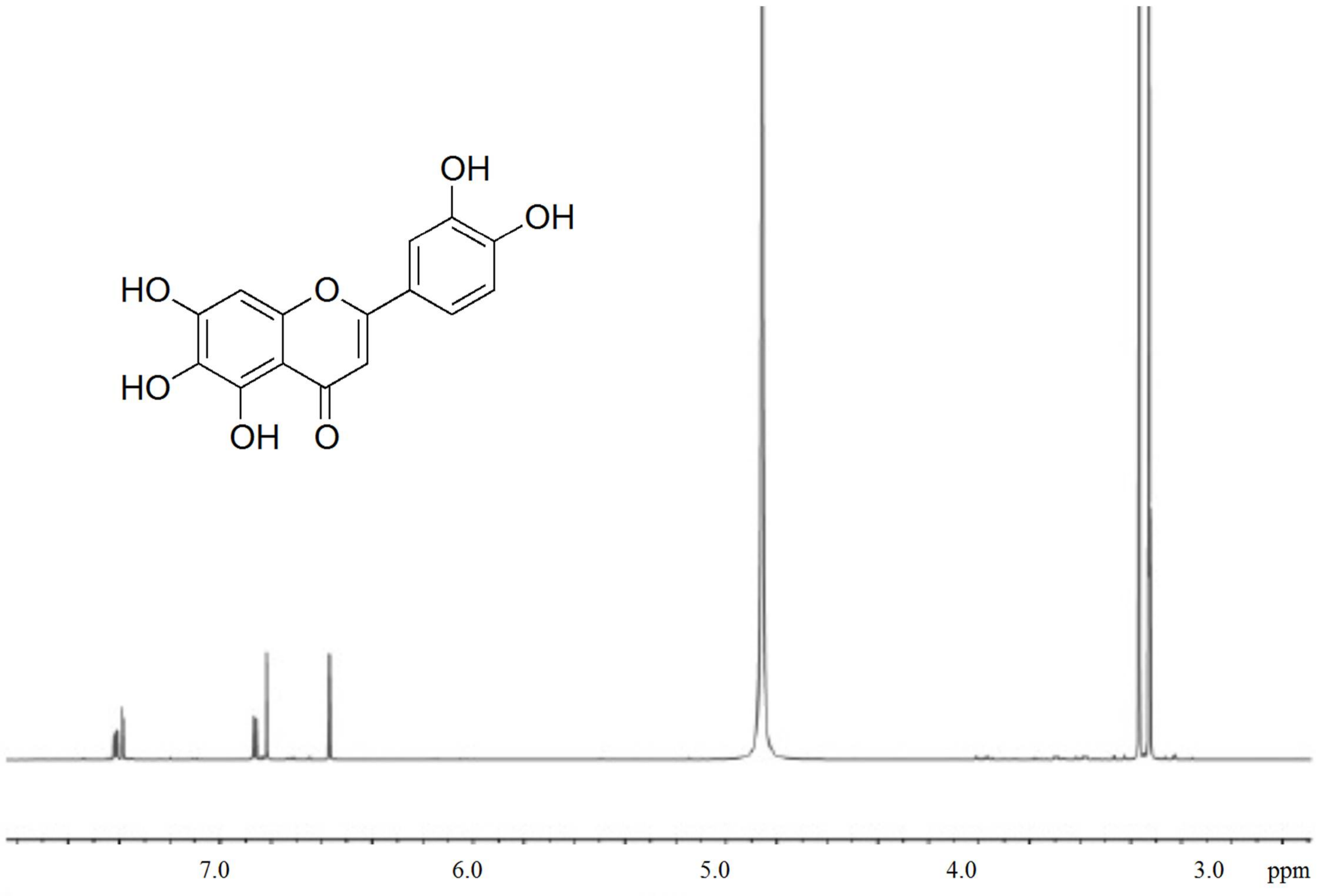

Figure S6. ^{13}C NMR spectra of 6-Hydroxyluteolin (compound **3**, 175 MHz, CD_3OD)

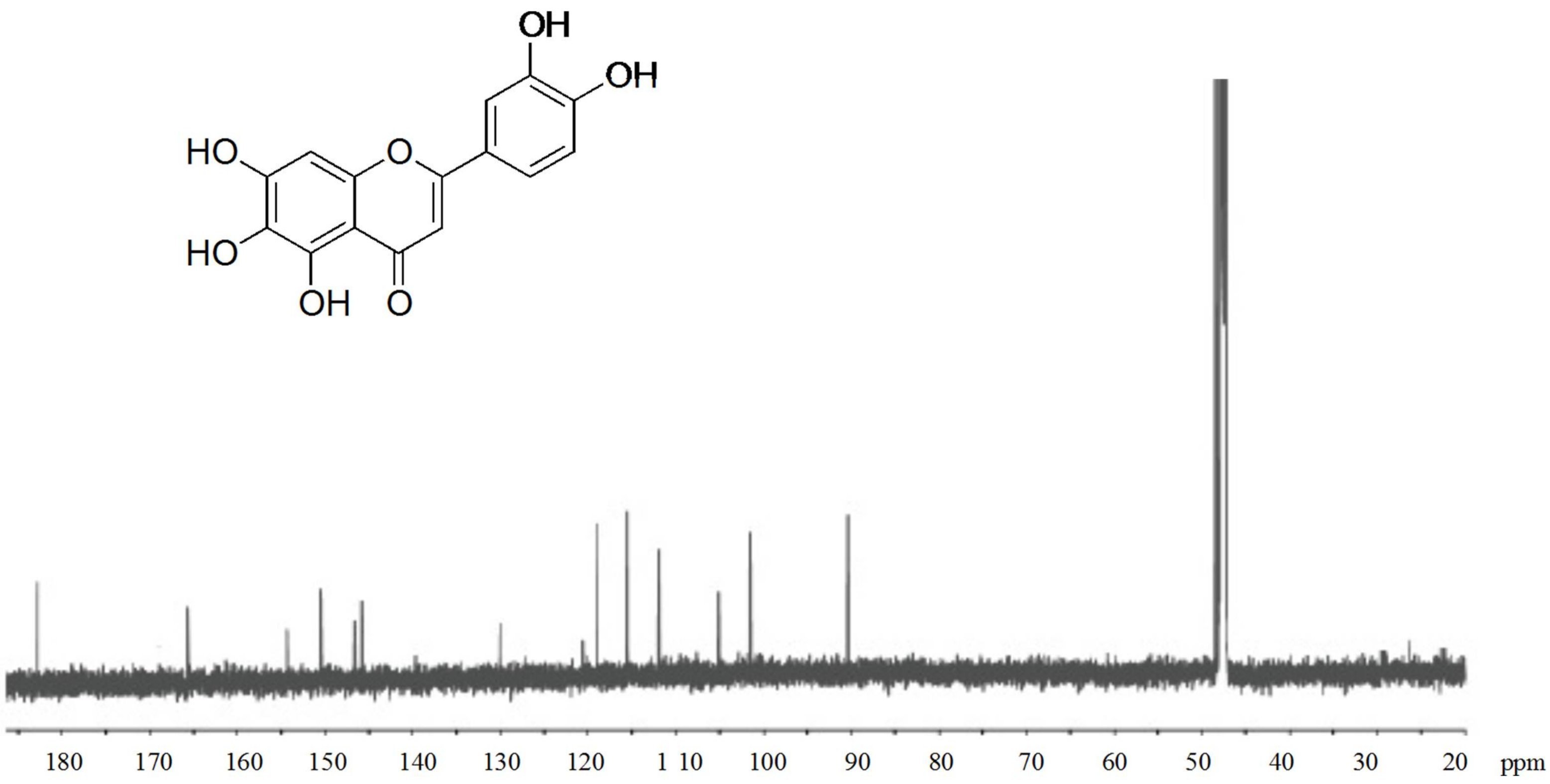

Figure S7. ^{1}H NMR spectrum of Rutin (compound **4**, 400 MHz, CD_3OD)

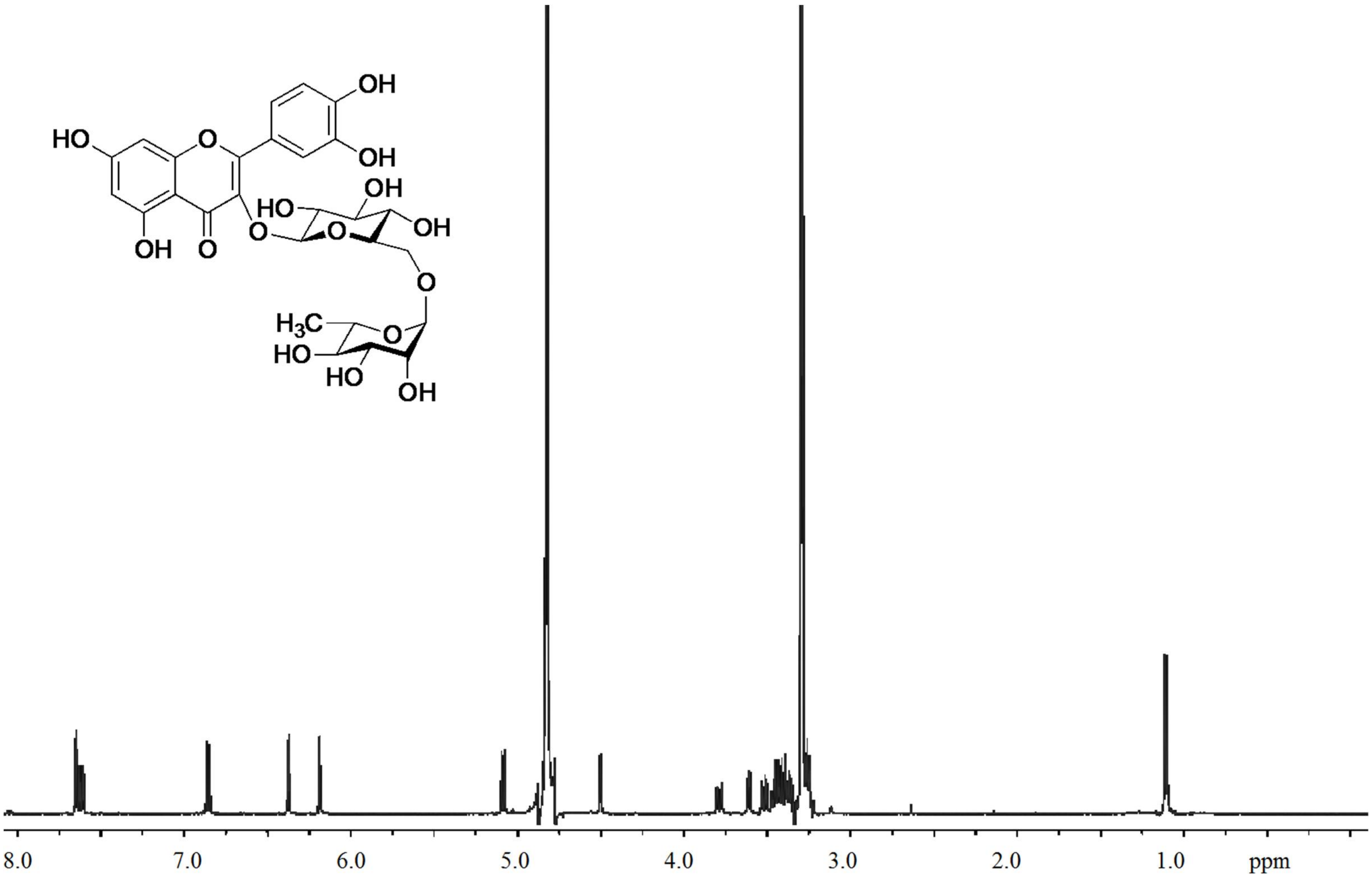

Figure S8. ^{1}H NMR spectrum of Rosmarinic acid (compound **5**, 700 MHz, CD_3OD)

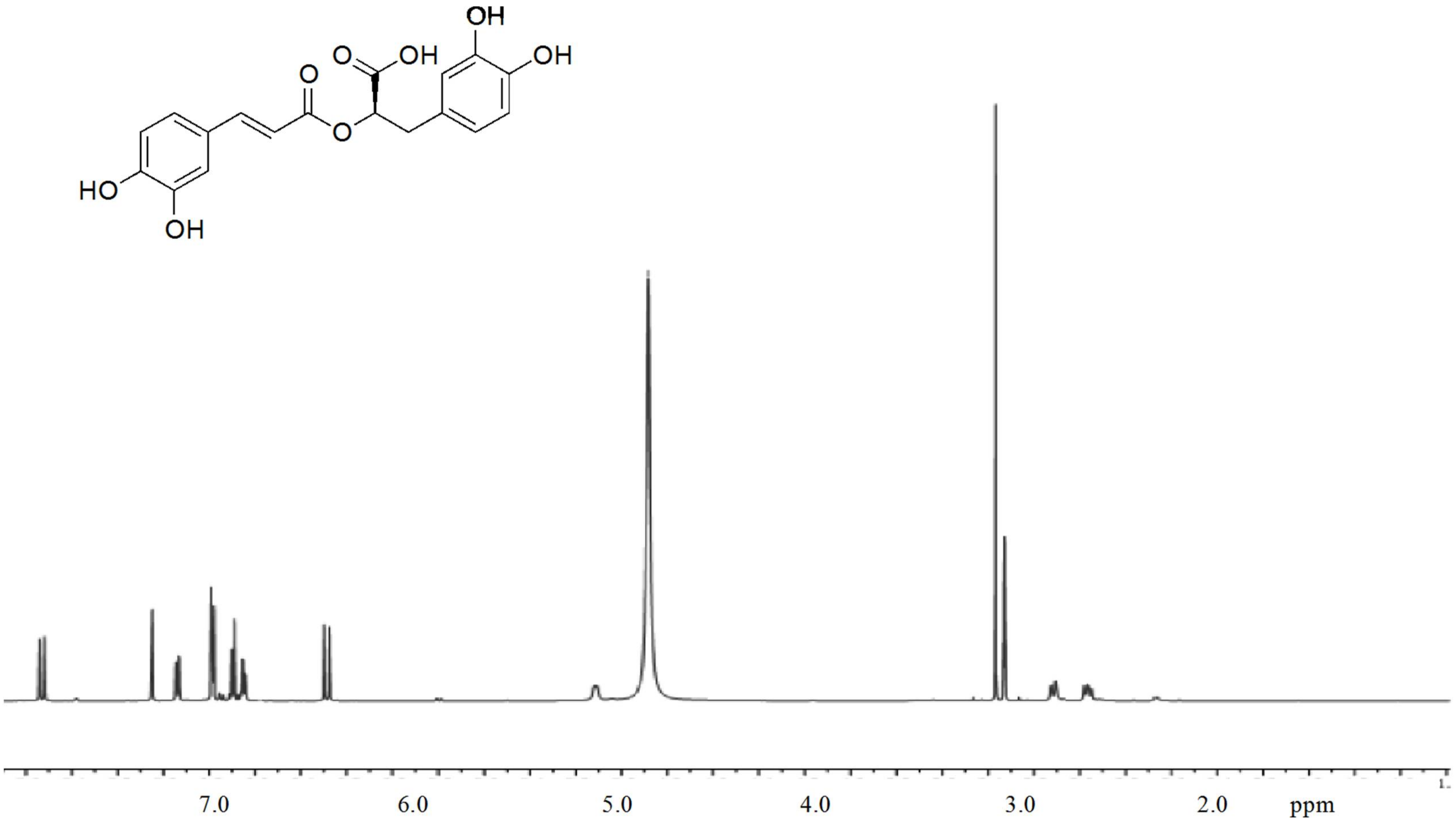

Figure S9. ^{13}C NMR spectrum of Rosmarinic acid (compound **5**, 175 MHz, CD_3OD)

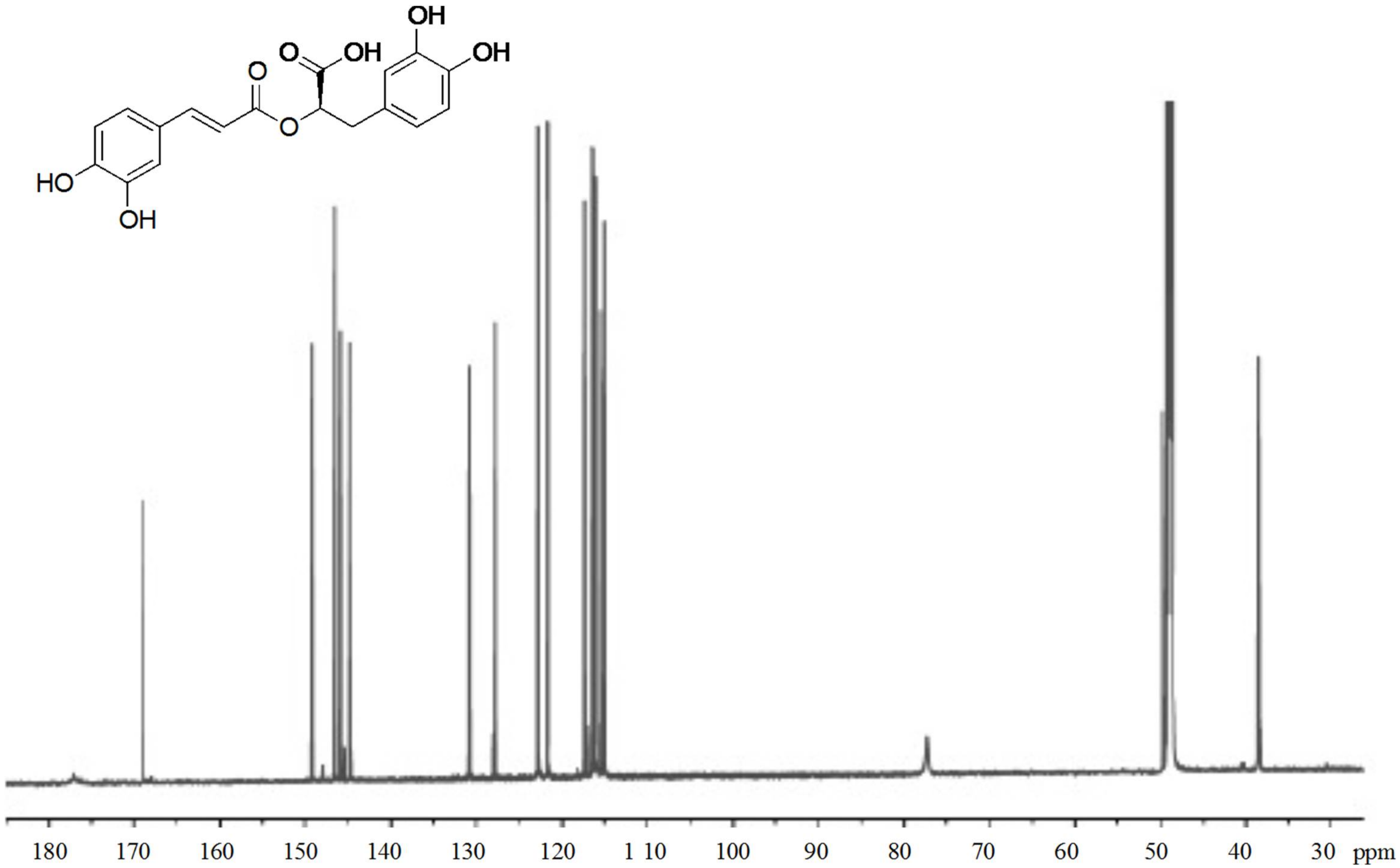

Figure S10. ^{1}H NMR spectrum of Isoquercitrin (compound **6**, 700 MHz, CD_3OD)

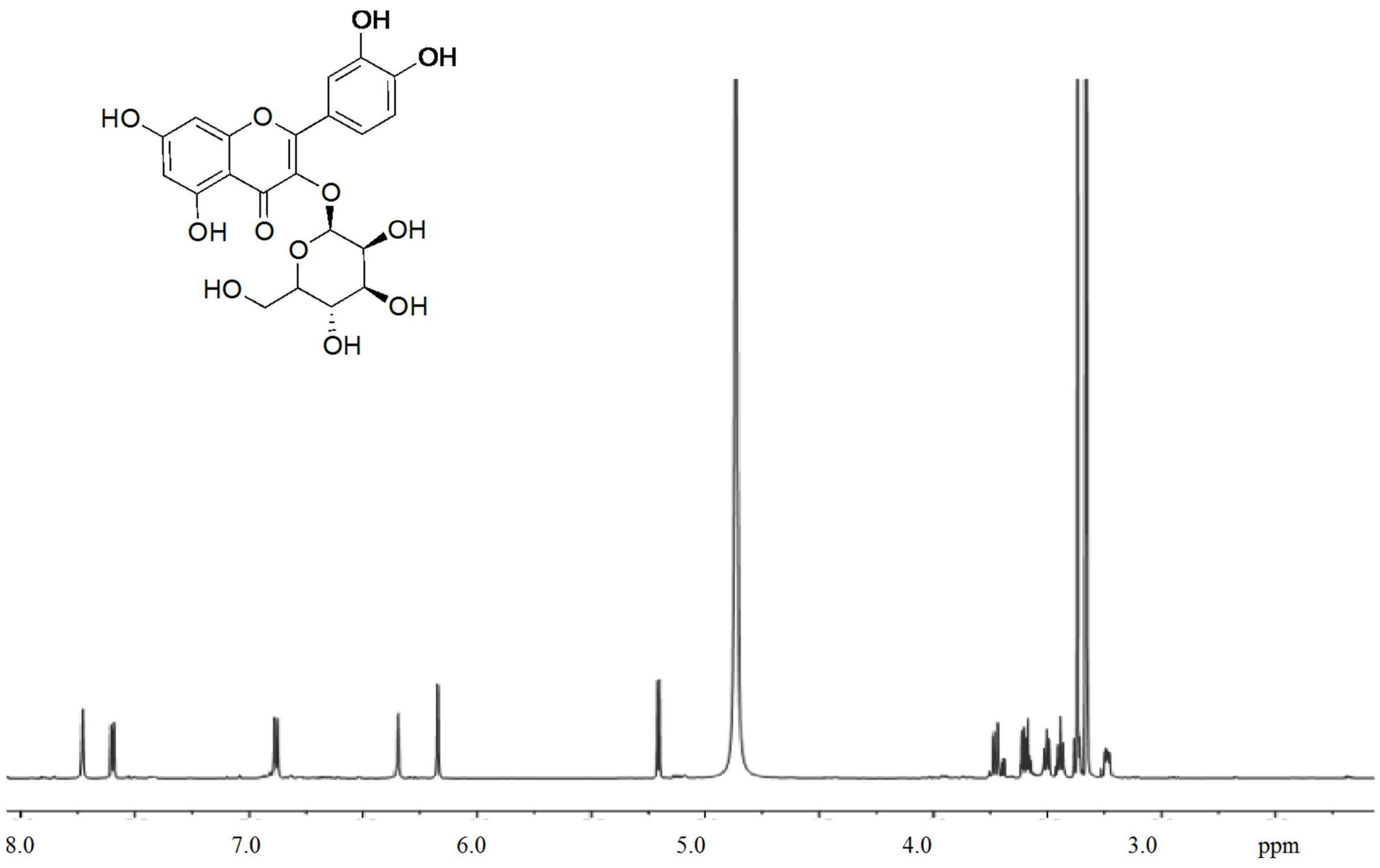

Figure S11. ^{13}C NMR spectrum of Isoquercitrin (compound **6**, 175 MHz, CD_3OD)

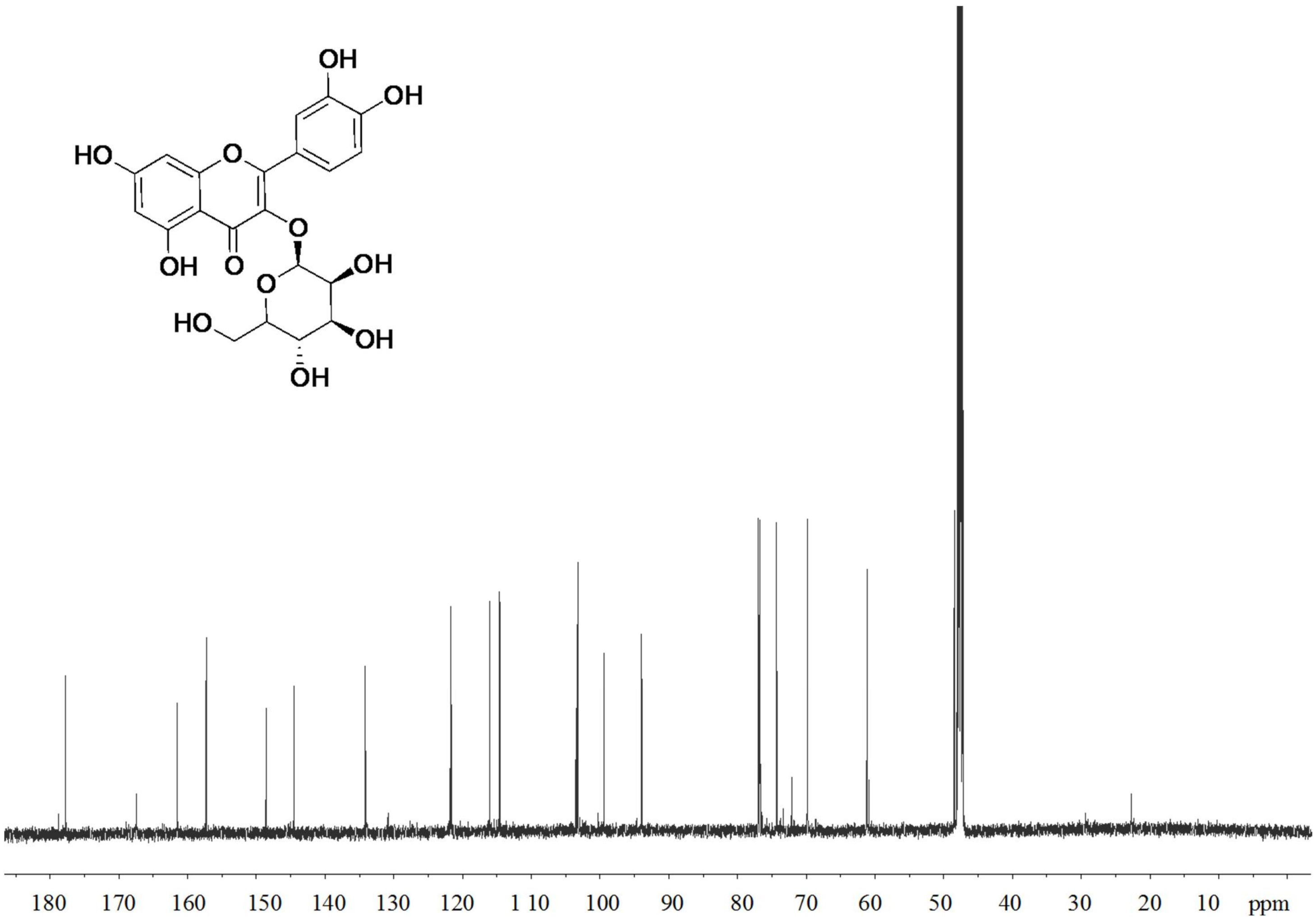

Figure S12. IR spectrum of Amarisolide G (compound **8a**,**b**)

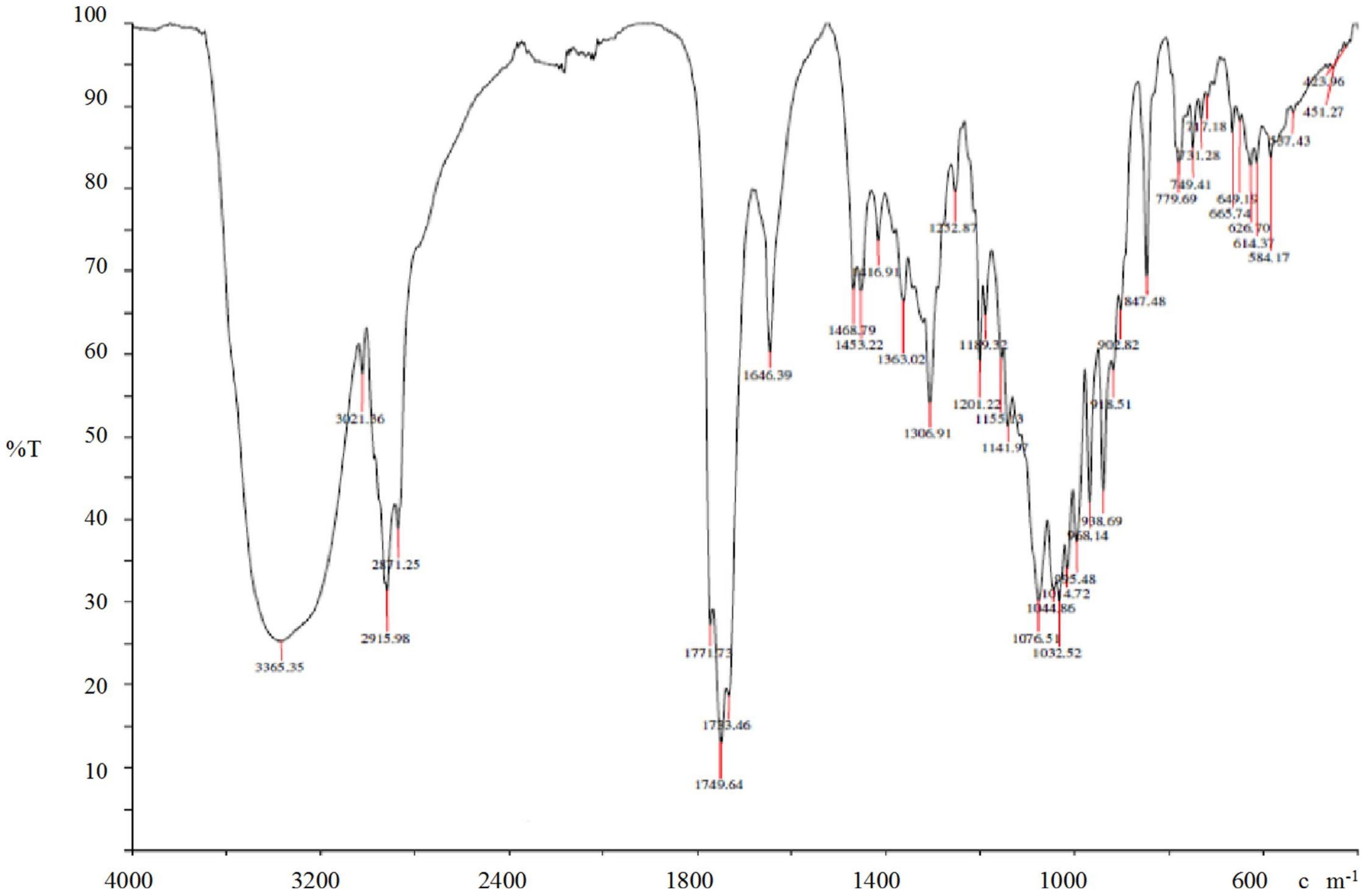

Figure S13. ^{1}H NMR spectrum of Amarisolide G (compound **8a**,**b**, 700 MHz, DMSO-d_6)

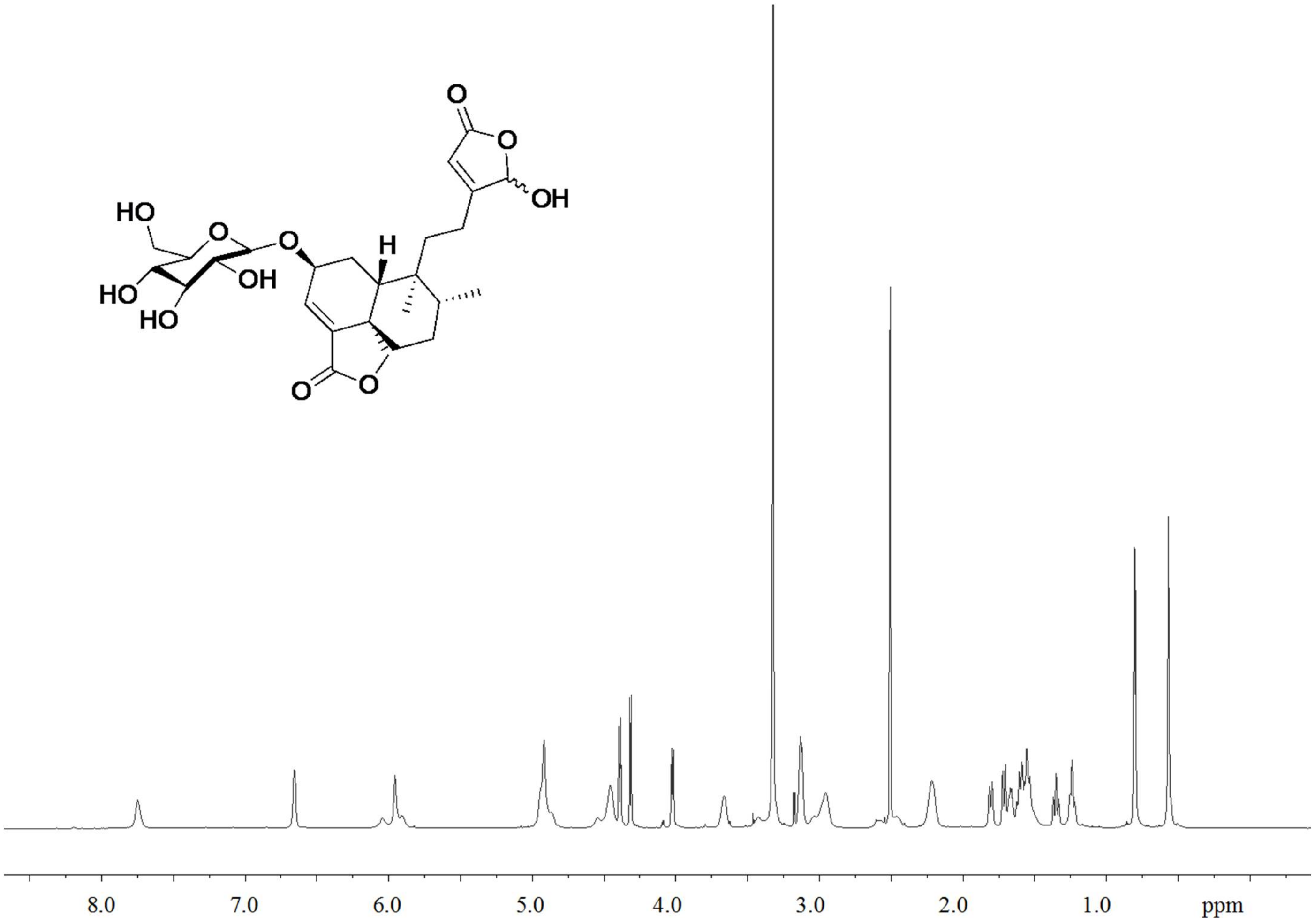

Figure S14. ^{13}C NMR spectrum of Amarisolide G (compound **8a**,**b**, 175 MHz, DMSO-d_6)

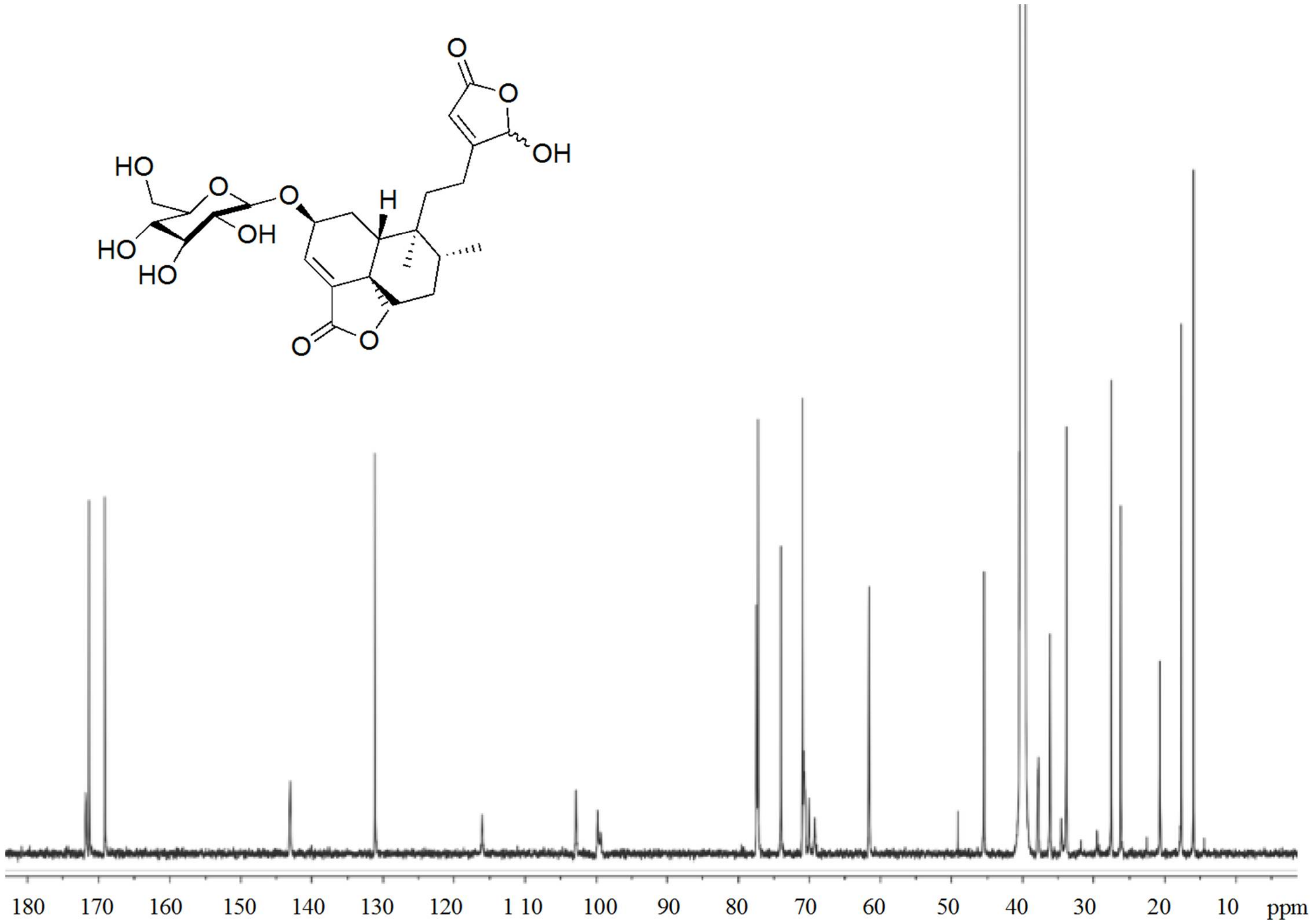

Figure S15. ^1H-^1H COSY spectrum of Amarisolide G (compound **8a**,**b**)

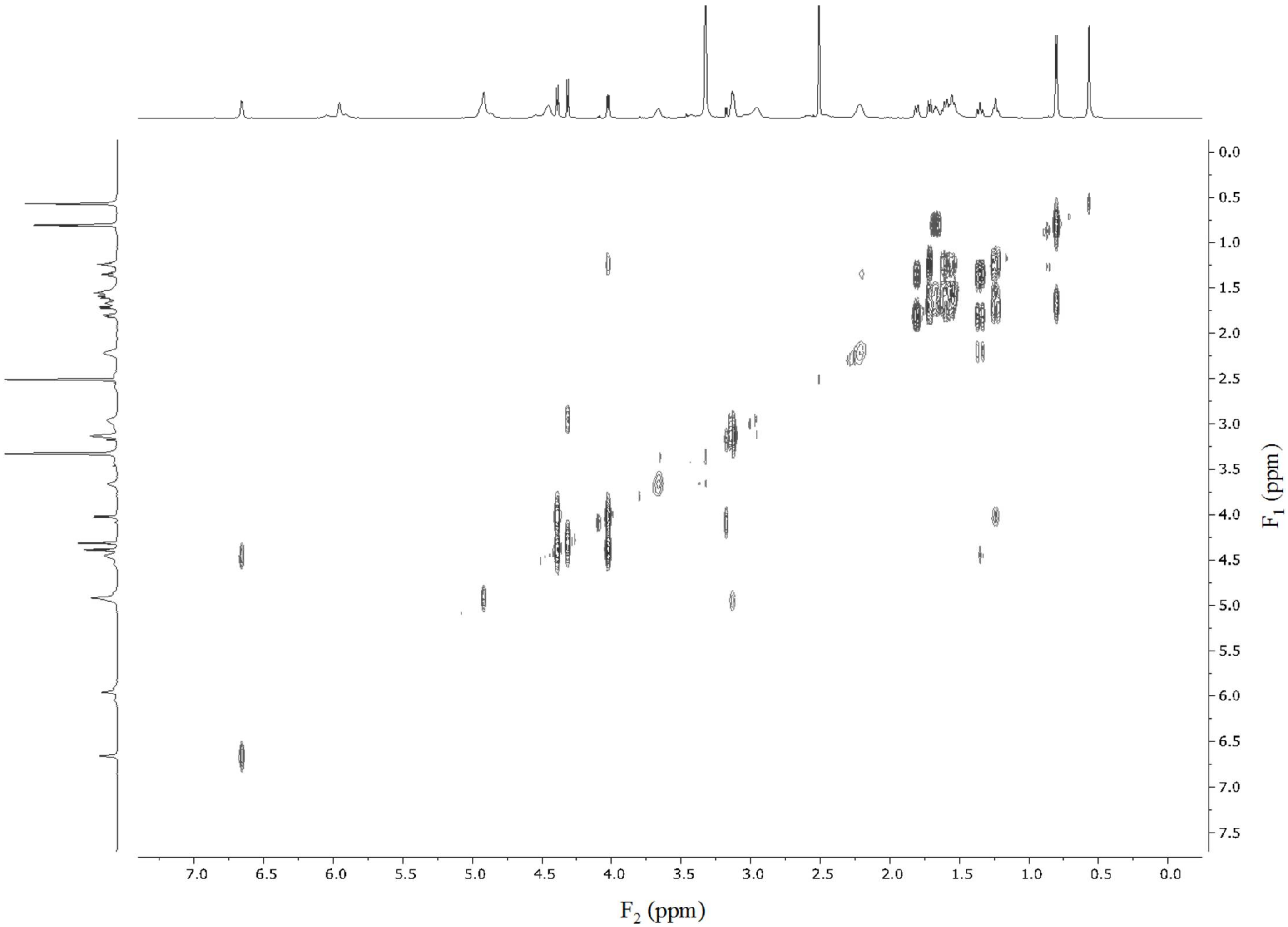

Figure S16. ^{1}H-^{13}C HSQC spectrum of Amarisolide G (compound **8a**,**b**)

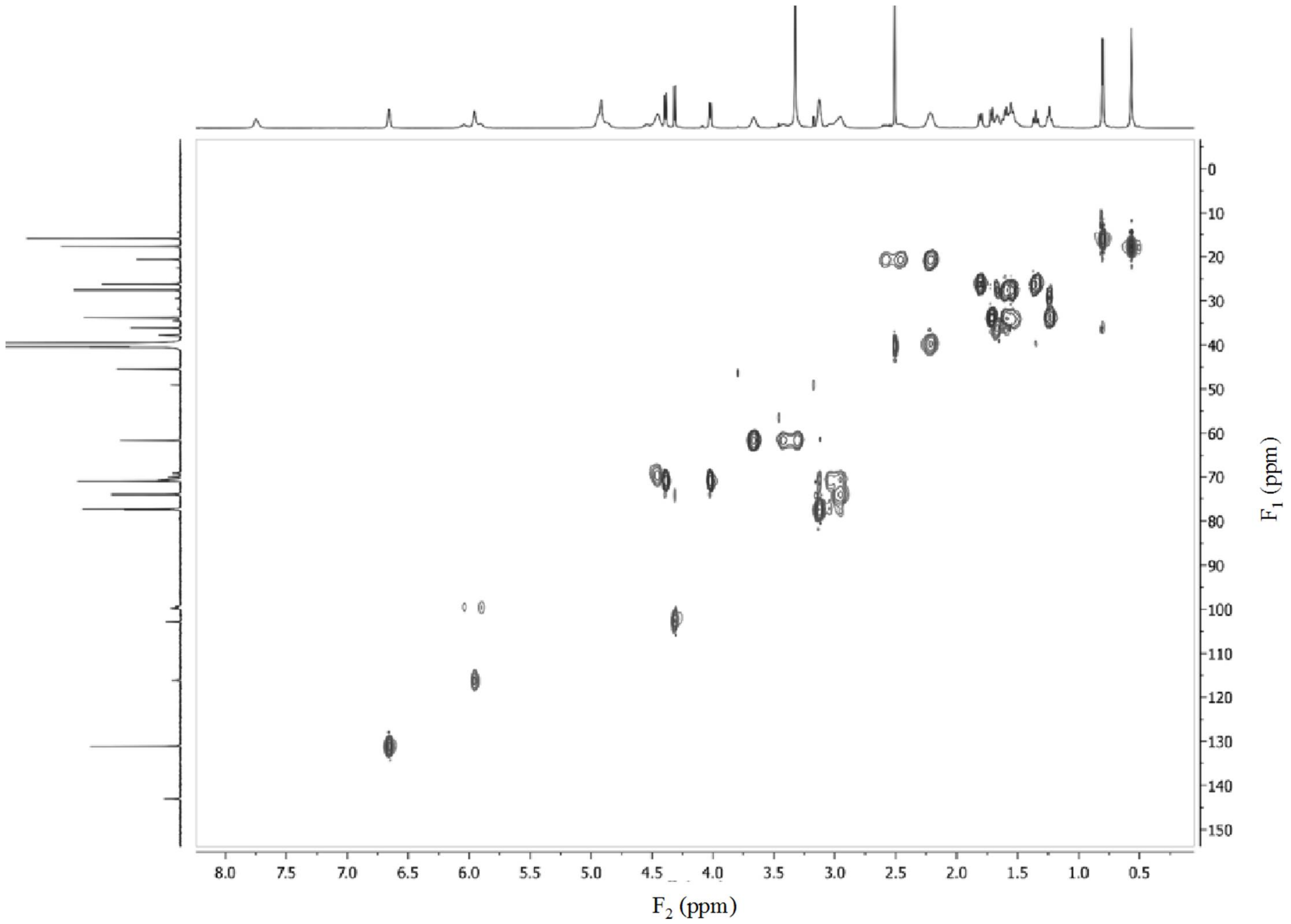

Figure S17. ^{1}H-^{13}C HMBC spectrum of Amarisolide G (compound **8a**,**b**)

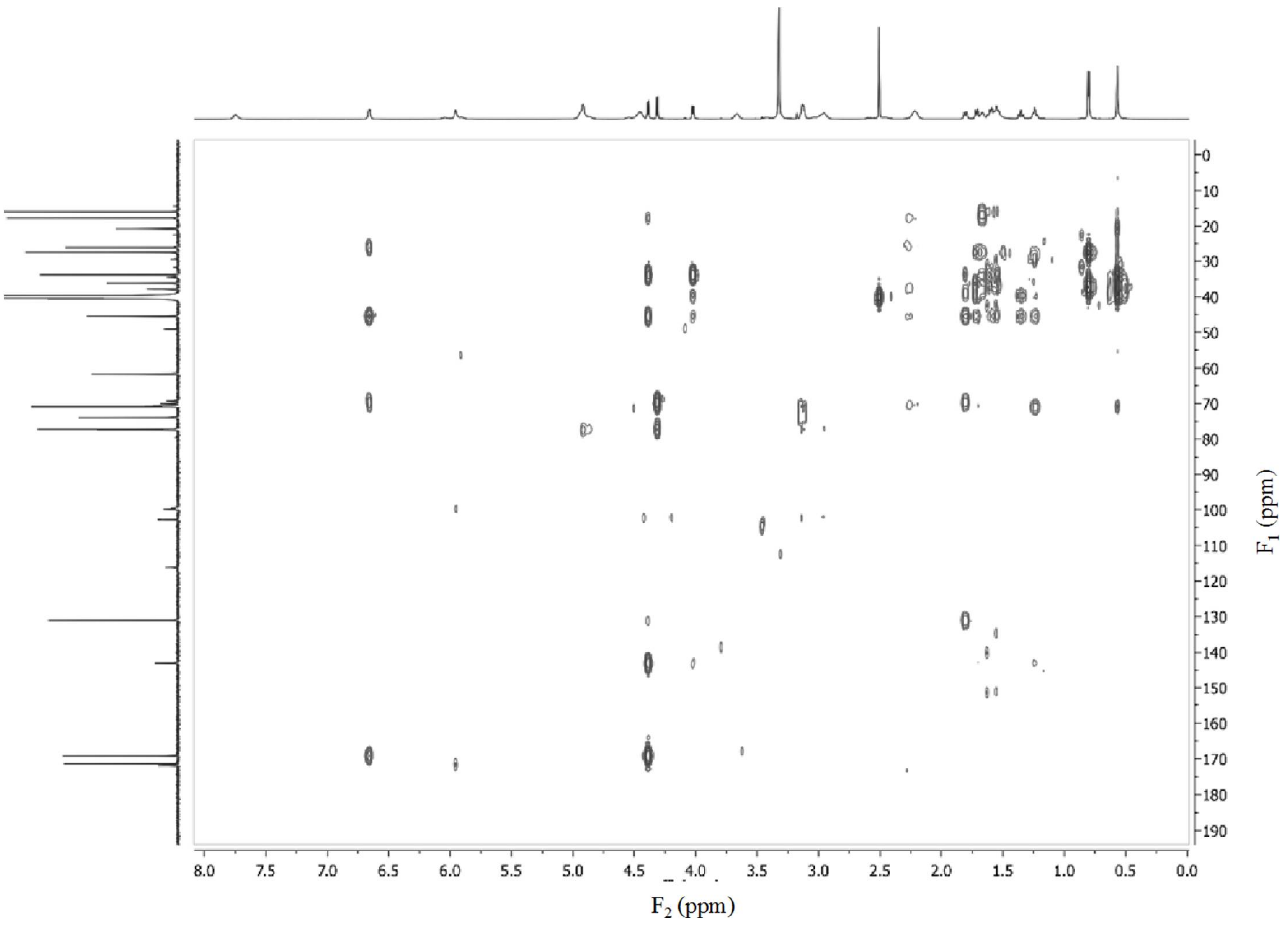

Figure S18. TOCSY spectrum of Amarisolide G (compound **8a**,**b**)

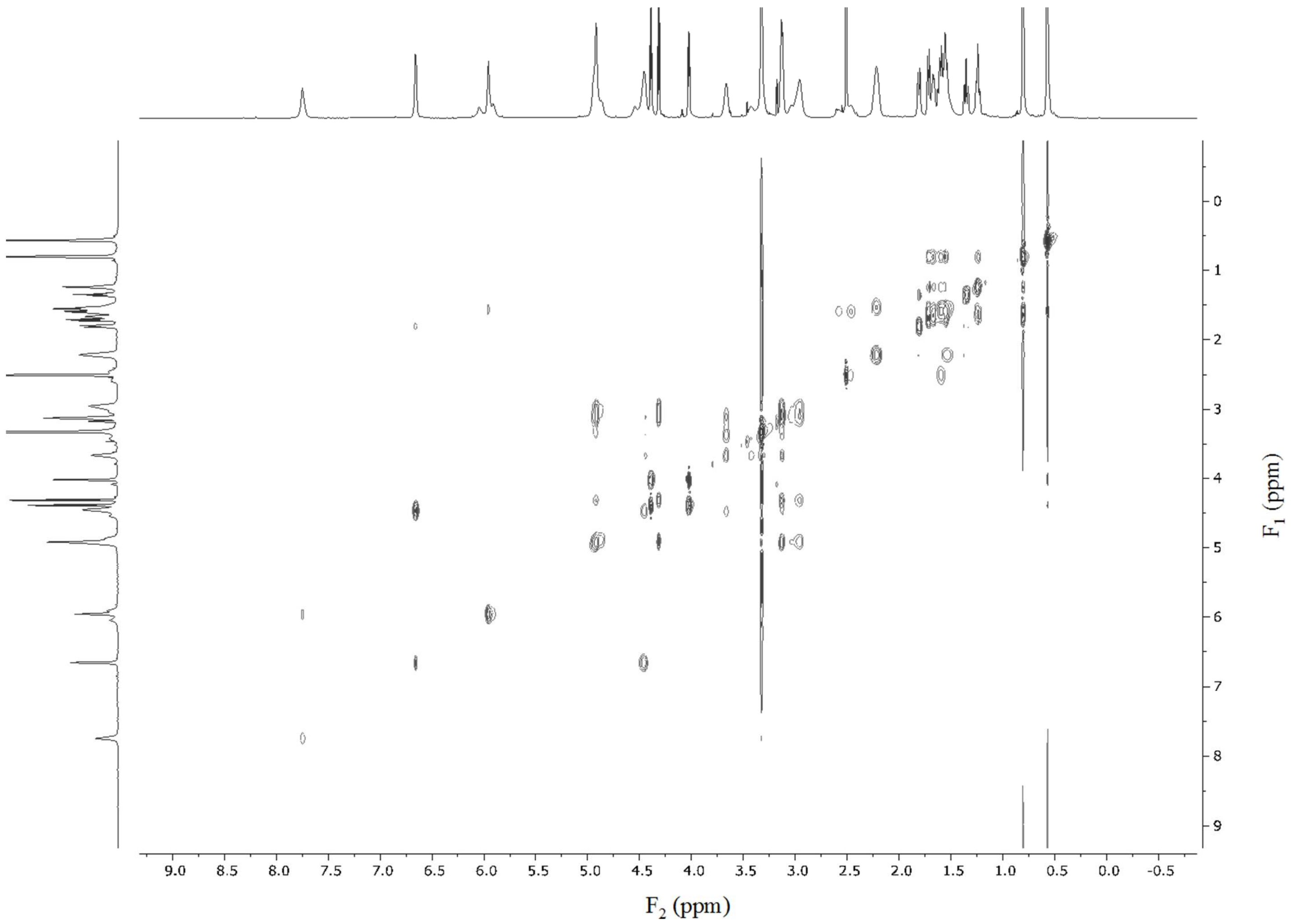

Figure S19. Total ion current chromatogram of the essential oil of *S. amarissima* (Sa-Batch 1). Peak identification: **10**, (*E*)-Pinocarvyl acetate; **11**, δ-elemene; **12**, α-bourbonene; **13**, β-caryophyllene; **14**, α-caryophyllene; **15**, germacrene D; **16**, β-selinene; **17**, spathulenol.

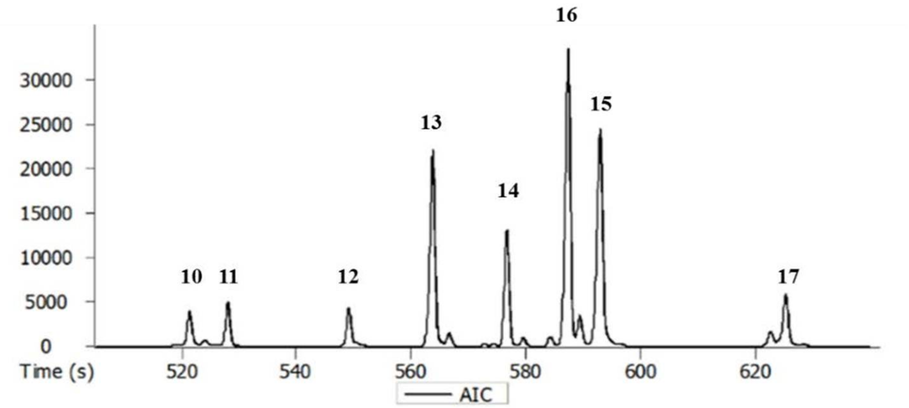

Figure S20. Total ion current chromatogram of the essential oil of *S. amarissima* (Sa-Batch 1). Peak identification: **9**, 3-Methoxy-*p*-cymene; **10**, (*E*)-pinocarvyl acetate; **12**, α-bourbonene; **13**, β-caryophyllene; **14**, α-caryophyllene; **15**, germacrene D; **16**, β-selinene; **17**, spathulenol.

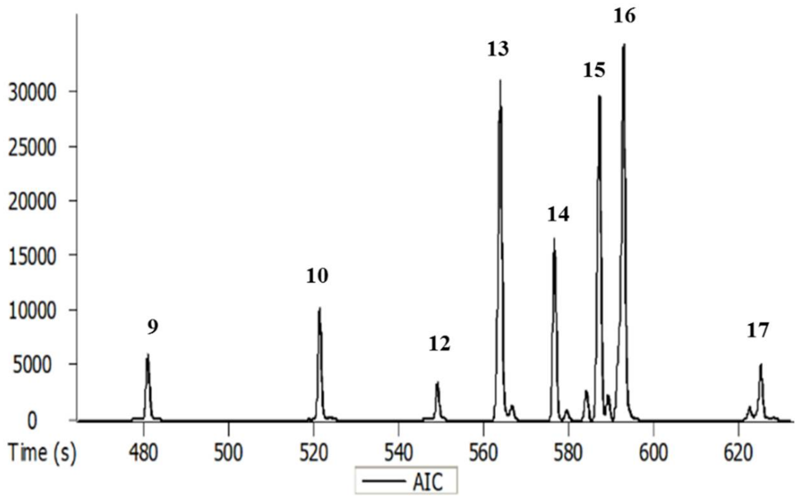

Figure S21. EIMS spectrum of 3-Methoxy-*p*-cymene (compound **9**)

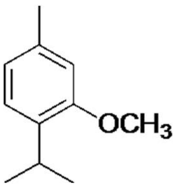

Similarity	T_R (s)	R_I (experimental)	R_I (literature)	Area (%)	Formula	Molecular Weight
978	480.894	1219	1215	4.40	$C_{11}H_{16}O$	164

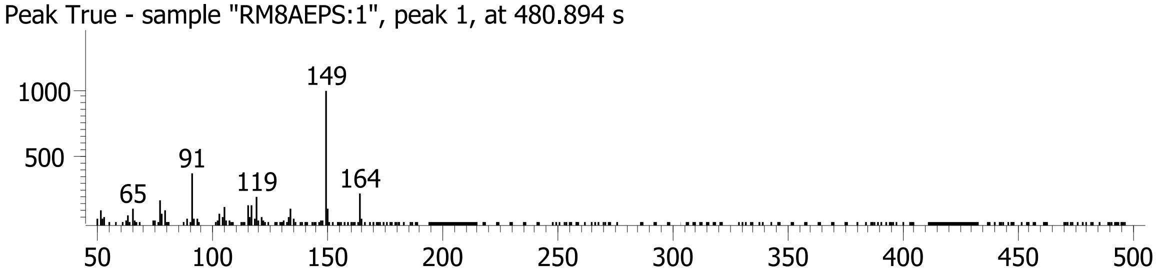

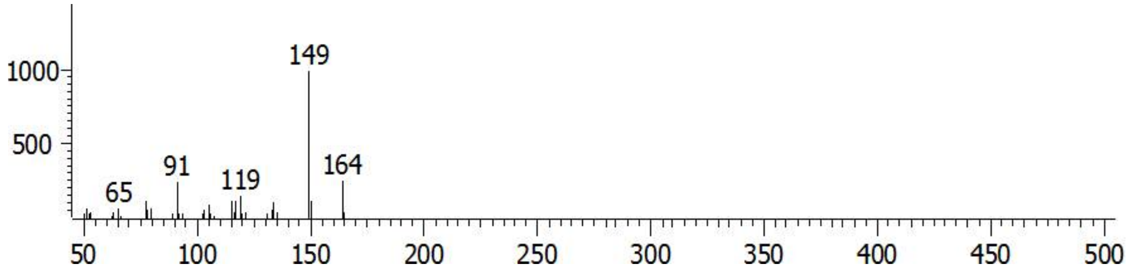

Figure S22. EIMS spectrum of (*E*)-Pinocarvyl acetate (compound **10**)

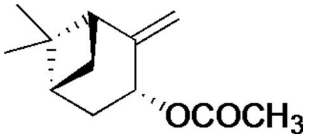

Similarity	T_R (s)	R_I (experimental)	R_I (literature)	Area (%)	Formula	Molecular Weight
910	521.365	1313	1312	5.98	$C_{12}H_{18}O_2$	194

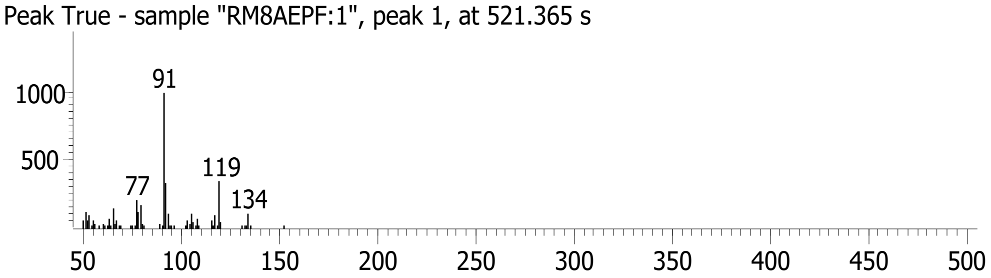

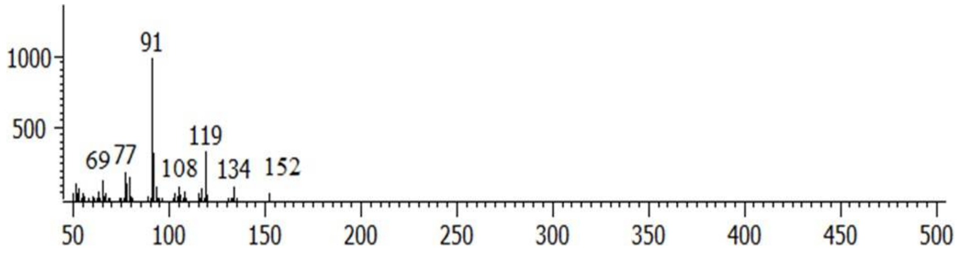

Figure S23. EIMS spectrum of δ-Elemene (compound **11**)

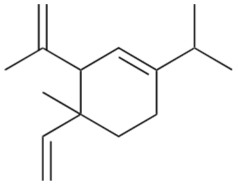

Similarity	T_R (s)	R_I (experimental)	R_I (literature)	Area (%)	Formula	Molecular Weight
900	528.065	1329	1326	1.99	$C_{15}H_{24}$	204.35

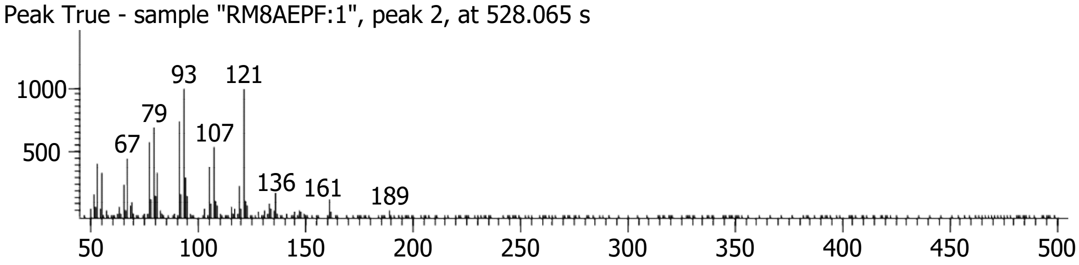

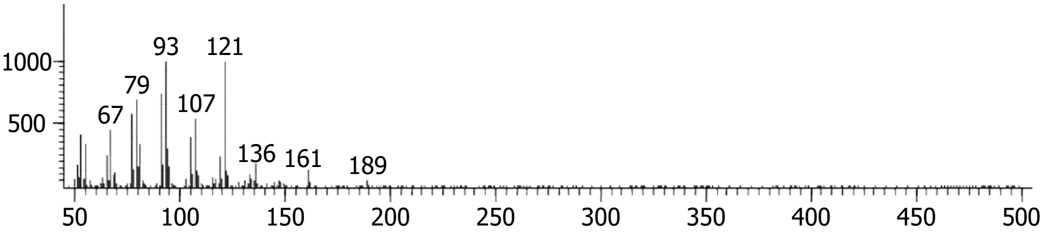

Figure S24. EIMS spectrum of α-Bourbonene (compound **12**)

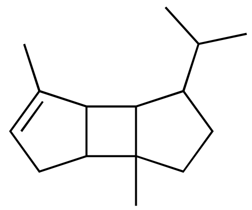

Similarity	T_R (s)	R_I (experimental)	R_I (literature)	Area (%)	Formula	Molecular Weight
900	549.165	1378	1374	4.24	$C_{15}H_{24}$	204.35

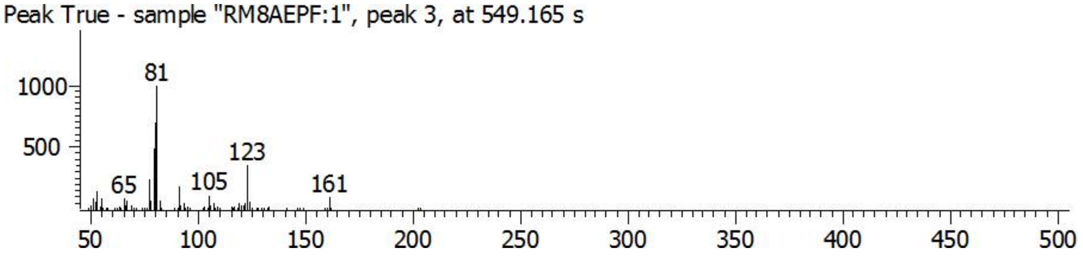

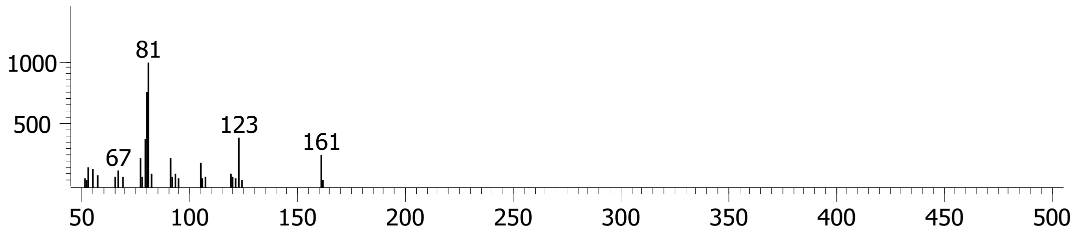

Figure S25. EIMS spectrum of β-Caryophyllene (compound **13**)

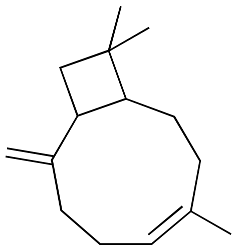

Similarity	T_R (s)	R_I (experimental)	R_I (literature)	Area (%)	Formula	Molecular Weight
933	563.765	1413	1414	15.05	$C_{15}H_{24}$	204.35

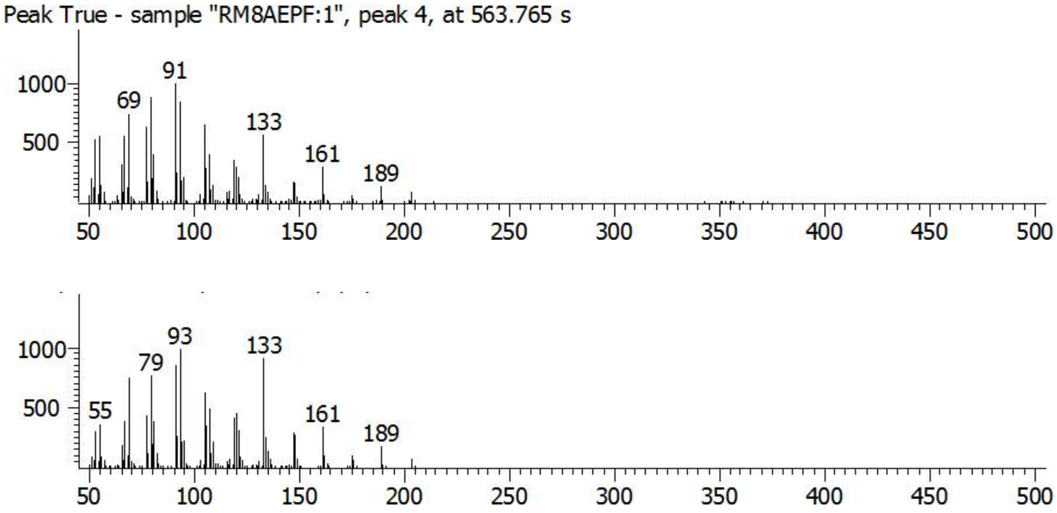

Figure S26. EIMS spectrum of α-Caryophyllene (compound **14**)

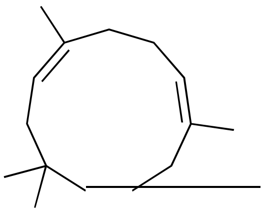

Similarity	T_R (s)	R_I (experimental)	R_I (literature)	Area (%)	Formula	Molecular Weight
910	576.665	1447	1446	7.68	$C_{15}H_{24}$	204.35

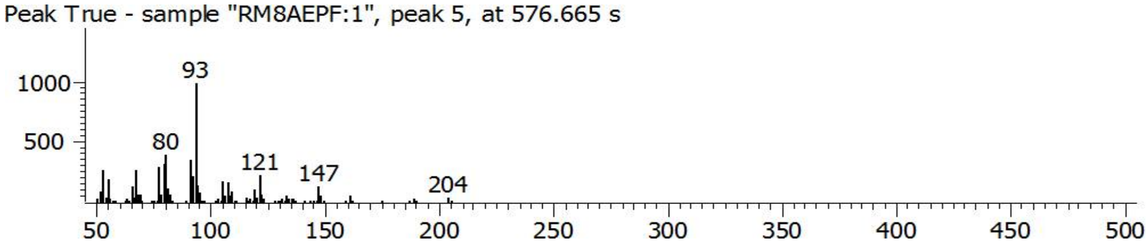

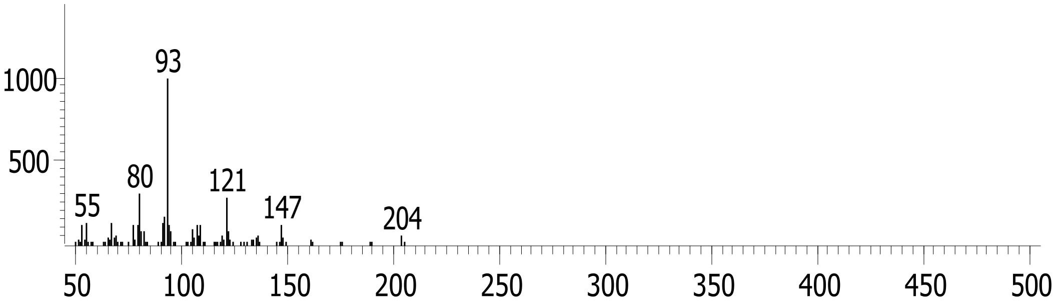

Figure S27. EIMS spectrum of Germacrene D (compound **15**)

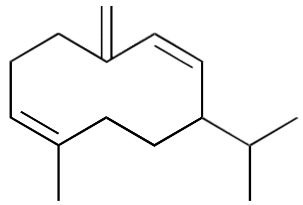

Similarity	T_R (s)	R_I (experimental)	R_I (literature)	Area (%)	Formula	Molecular Weight
910	587.365	1476	1476	25.09	$C_{15}H_{24}$	204.35

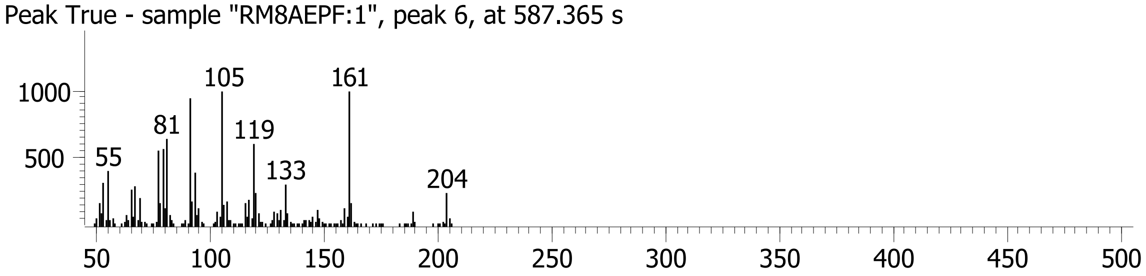

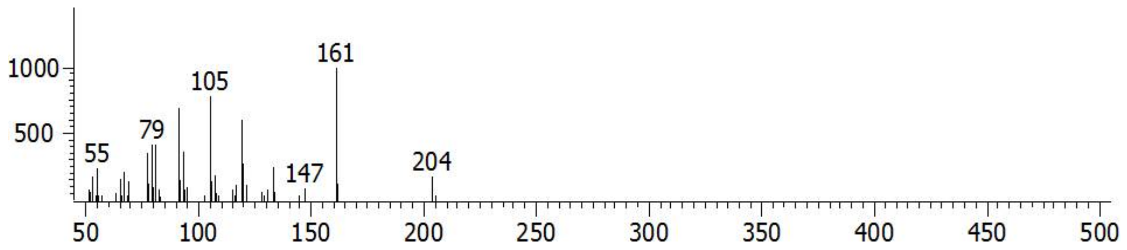

Figure S28. EIMS spectrum of β-Selinene (compound **16**)

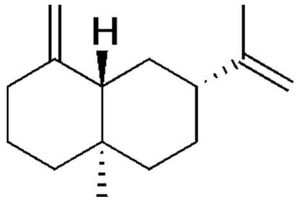

Similarity	T_R (s)	R_I (experimental)	R_I (literature)	Area (%)	Formula	Molecular Weight
980	592.965	1491	1489	28.35	$C_{15}H_{24}$	204.35

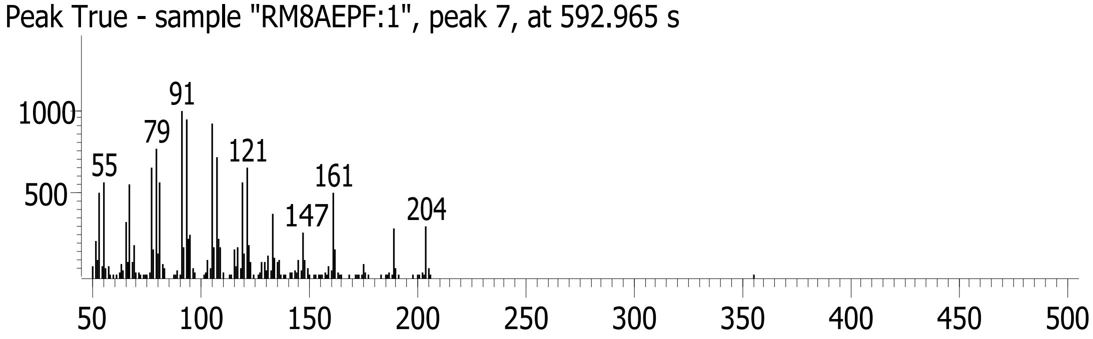

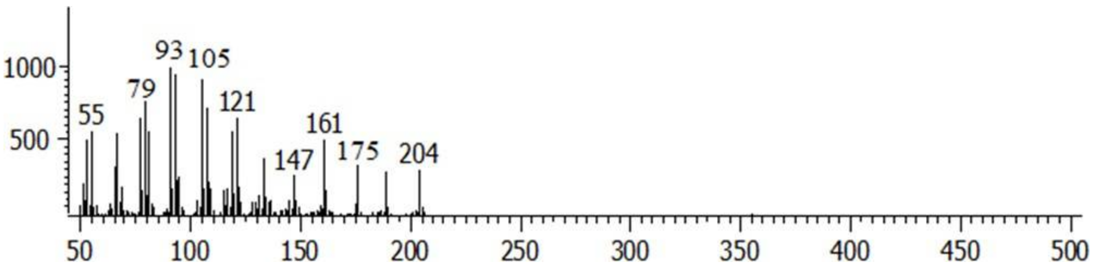

Figure S29. EIMS spectrum of Spathulenol (compound **17**)

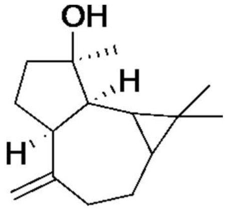

Similarity	T_R (s)	R_I (experimental)	R_I (literature)	Area (%)	Formula	Molecular Weight
999	625.265	1576	1576	11.59	$C_{15}H_{24}O$	220

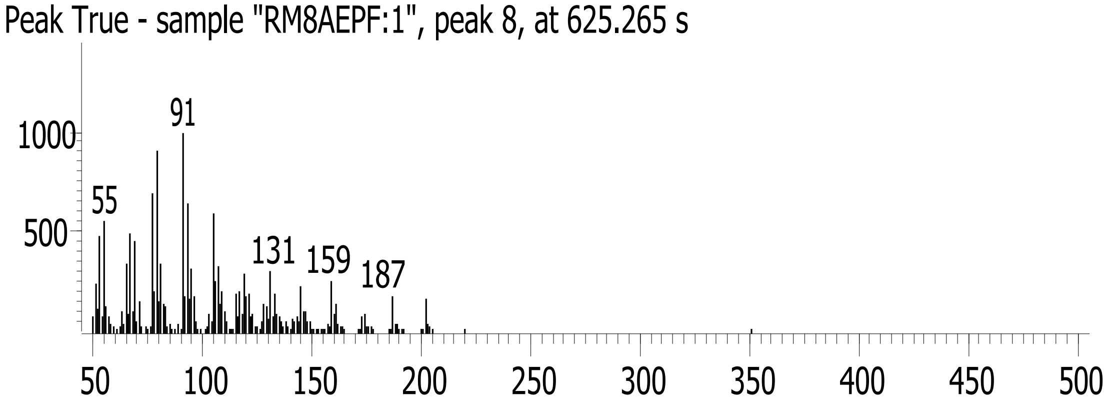

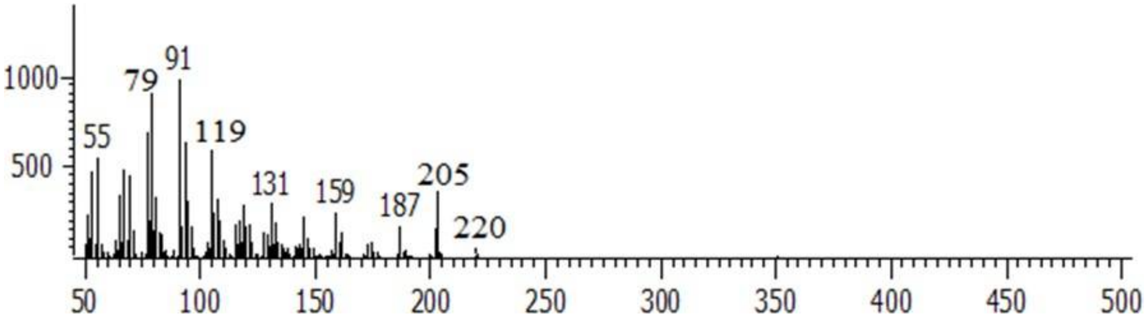

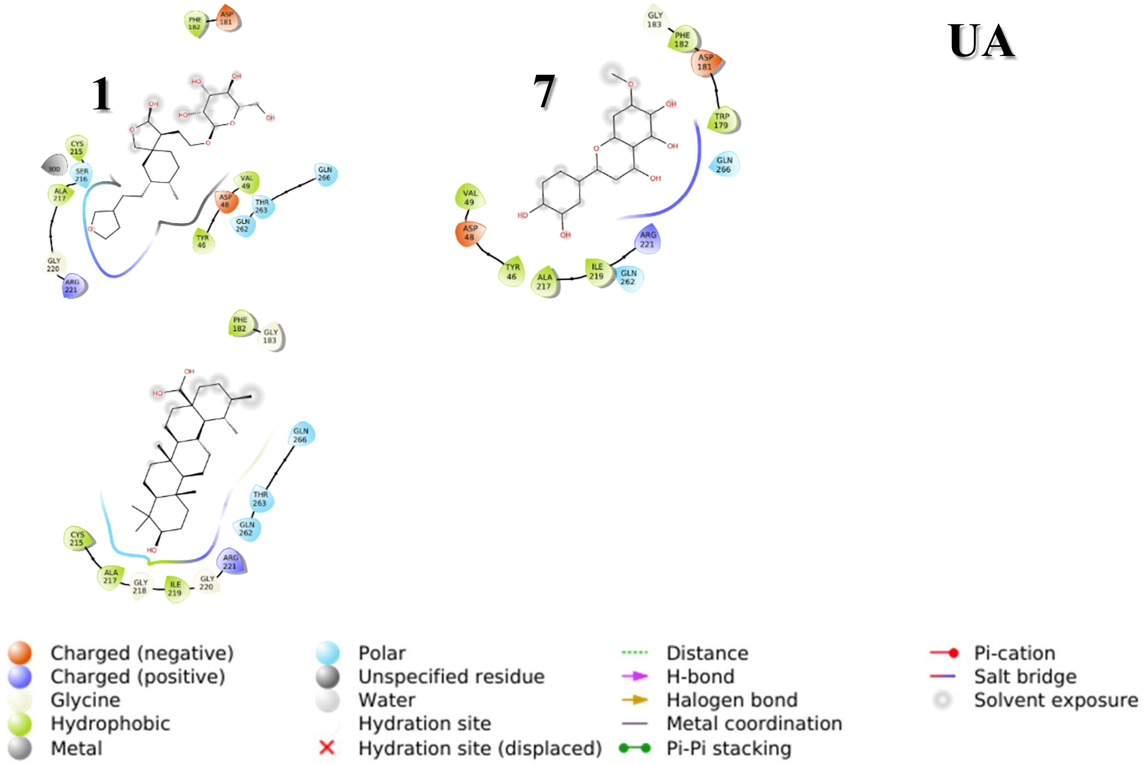

Figure S30. Residues of interaction to 4 Å of compounds **1**,**7** and **UA** at the catalytic site of PTP1B.

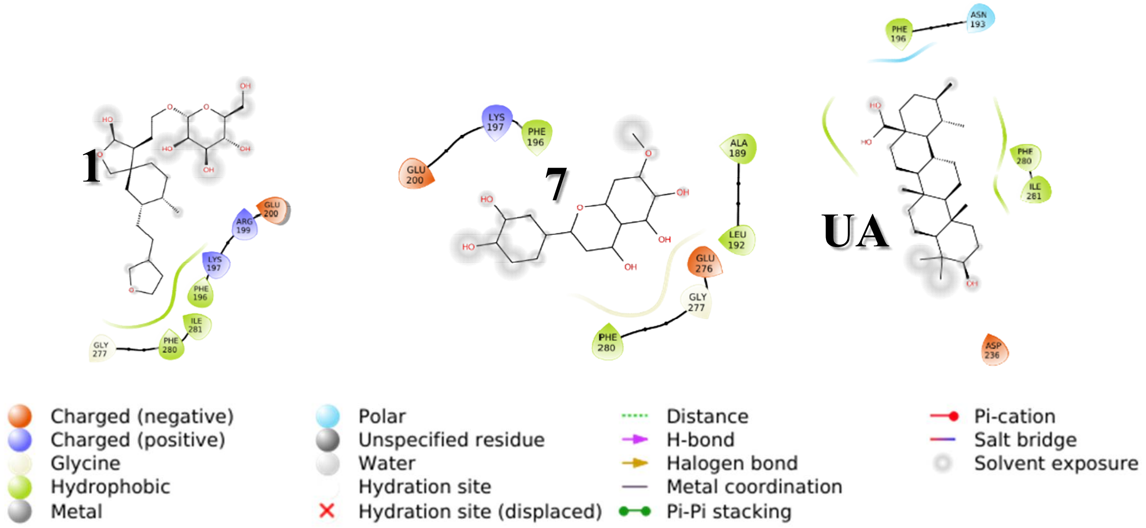

Figure S31. Residues of interaction to 4 Å of compounds **1**, **7** and **UA** at the allosteric site of PTP1B.

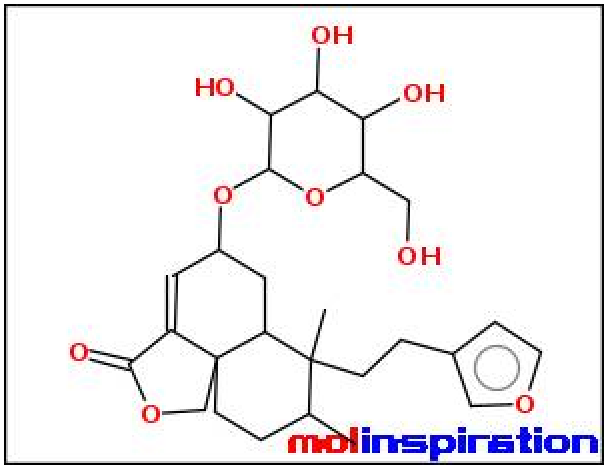

Molinspiration bioactivity score v2018.03
GPCR ligand
0.49
Ion channel modulator
0.36
Kinase inhibitor -
0.08
Nuclear receptor ligand
0.48
Protease inhibitor
0.28
Enzyme inhibitor
0.71

Get data as text (for copy / paste).

Get 3D geometry BETA

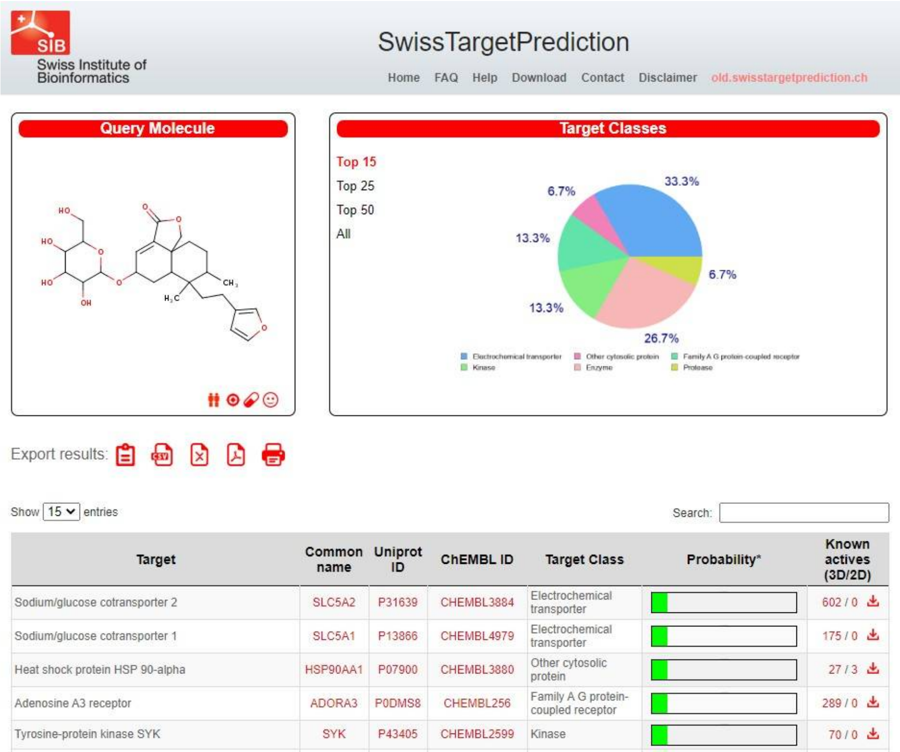

Target	Common name	Uniprot ID	ChEMBL ID	Target Class	Probability*	Known actives (3D/2D)
Sodium/glucose cotransporter 2	SLC5A2	P31639	CHEMBL3884	Electrochemical transporter		602 / 0
Sodium/glucose cotransporter 1	SLC5A1	P13866	CHEMBL4979	Electrochemical transporter		175 / 0
Heat shock protein HSP 90-alpha	HSP90AA1	P07900	CHEMBL3880	Other cytosolic protein		27 / 3
Adenosine A3 receptor	ADORA3	P0DMS8	CHEMBL256	Family A G protein-coupled receptor		289 / 0
Tyrosine-protein kinase SYK	SYK	P43405	CHEMBL2599	Kinase		70 / 0

Figure S32. Prediction of biological properties of compounds **1**, using servers Molinspiration and server Swiss TargetPrediction.

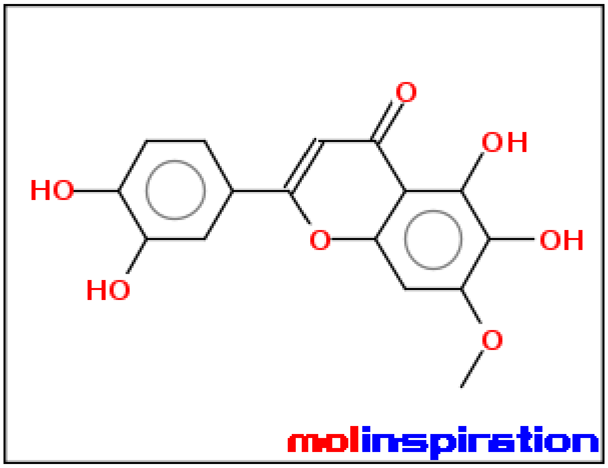

Molinspiration bioactivity score v2018.03
GPCR ligand -0.10
Ion channel modulator -0.24
Kinase inhibitor 0.24
Nuclear receptor ligand 0.18
Protease inhibitor -0.32
Enzyme inhibitor 0.19

Get data as text (for copy / paste).

Get 3D geometry BETA

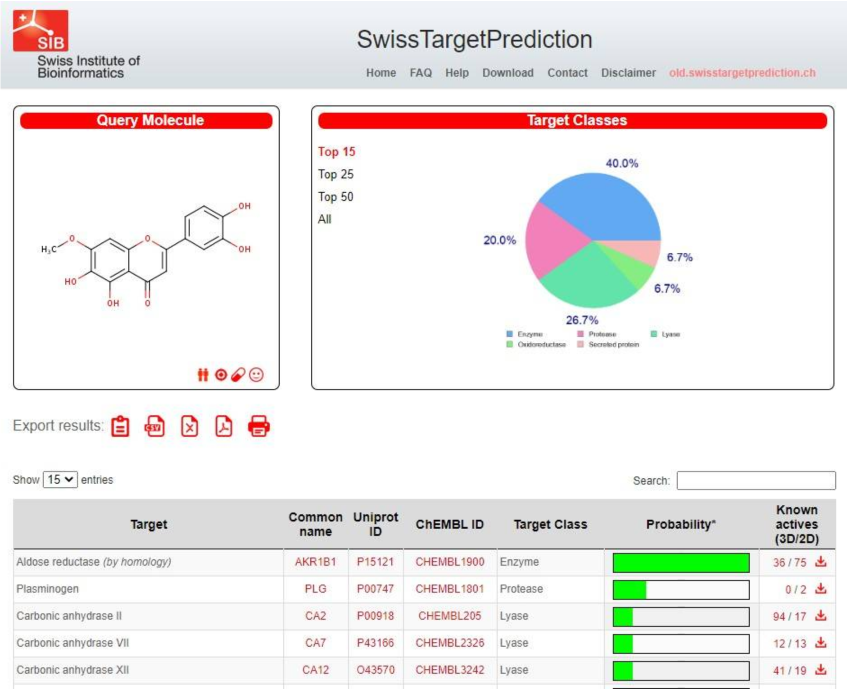

Figure S33. Prediction of biological properties of compounds **7**, using servers Molinspiration and server Swiss TargetPrediction.

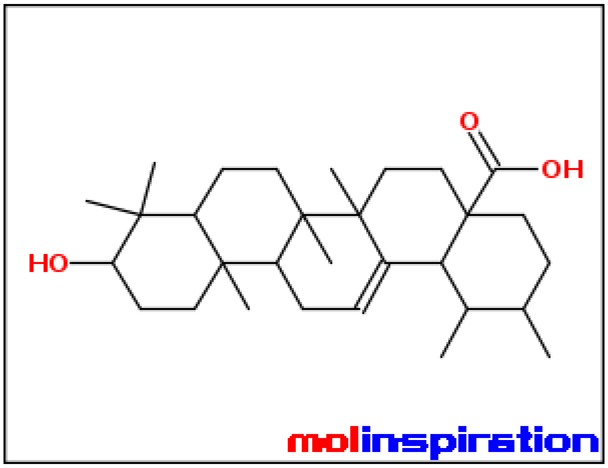

Molinspiration bioactivity score v2018.03

GPCR ligand 0.28

Ion channel modulator -0.03

Kinase inhibitor -0.50

Nuclear receptor ligand 0.89

Protease inhibitor 0.23

Enzyme inhibitor 0.69

Get data as text (for copy / paste).

Get 3D geometry BETA

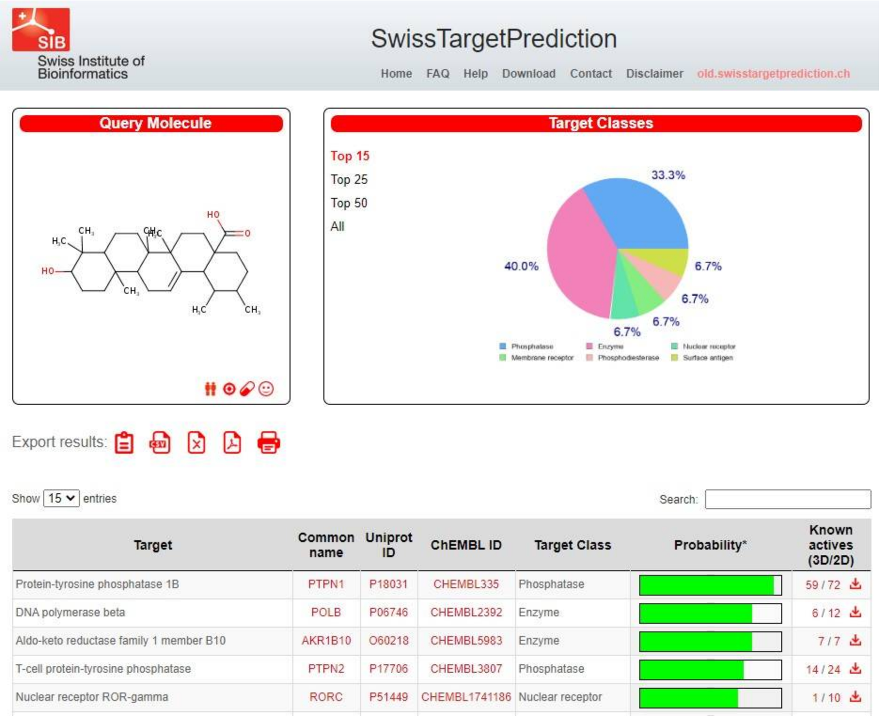

Target	Common name	Uniprot ID	ChEMBL ID	Target Class	Probability*	Known actives (3D/2D)
Protein-tyrosine phosphatase 1B	PTPN1	P18031	CHEMBL335	Phosphatase		59 / 72
DNA polymerase beta	POLB	P06746	CHEMBL2392	Enzyme		6 / 12
Aldo-keto reductase family 1 member B10	AKR1B10	O60218	CHEMBL5983	Enzyme		7 / 7
T-cell protein-tyrosine phosphatase	PTPN2	P17706	CHEMBL3807	Phosphatase		14 / 24
Nuclear receptor ROR-gamma	RORC	P51449	CHEMBL1741186	Nuclear receptor		1 / 10

Figure S34. Prediction of biological properties of compounds **AU**, using servers Molinspiration and server Swiss TargetPrediction.
